# Perioperative cerebrospinal fluid and plasma inflammatory markers after orthopedic surgery

**DOI:** 10.1186/s12974-016-0681-9

**Published:** 2016-08-30

**Authors:** Jan Hirsch, Susana Vacas, Niccolo Terrando, Miao Yuan, Laura P. Sands, Joel Kramer, Kevin Bozic, Mervyn M. Maze, Jacqueline M. Leung

**Affiliations:** 1 Department of Anesthesia and Perioperative Care, University of California San Francisco, 521 Parnassus Avenue, San Francisco, CA 94143-0648 USA; 2 Anesthesia Service, San Francisco VA Medical Center, Mail 129, 4150 Clement Street, San Francisco, CA 94121 USA; 3 Center of Gerontology, Virginia Tech University, 230 Grove Lane (0555), Blacksburg, VA 24061 USA; 4 Department of Neurology, Memory and Aging Center, University of California San Francisco, 1500 Owens St. #320, San Francisco, CA 94158 USA; 5 Department of Orthopedic Surgery, University of California San Francisco, 500 Parnassus Avenue, MU 320W, San Francisco, CA 94143-0728 USA

**Keywords:** Blood-brain barrier, Surgery, Delirium, Immune response

## Abstract

**Background:**

Postoperative delirium is prevalent in older patients and associated with worse outcomes. Recent data in animal studies demonstrate increases in inflammatory markers in plasma and cerebrospinal fluid (CSF) even after aseptic surgery, suggesting that inflammation of the central nervous system may be part of the pathogenesis of postoperative cognitive changes. We investigated the hypothesis that neuroinflammation was an important cause for postoperative delirium and cognitive dysfunction after major non-cardiac surgery.

**Methods:**

After Institutional Review Board approval and informed consent, we recruited patients undergoing major knee surgery who received spinal anesthesia and femoral nerve block with intravenous sedation. All patients had an indwelling spinal catheter placed at the time of spinal anesthesia that was left in place for up to 24 h. Plasma and CSF samples were collected preoperatively and at 3, 6, and 18 h postoperatively. Cytokine levels were measured using ELISA and Luminex. Postoperative delirium was determined using the confusion assessment method, and cognitive dysfunction was measured using validated cognitive tests (word list, verbal fluency test, digit symbol test).

**Results:**

Ten patients with complete datasets were included. One patient developed postoperative delirium, and six patients developed postoperative cognitive dysfunction. Postoperatively, at different time points, statistically significant changes compared to baseline were present in IL-5, IL-6, I-8, IL-10, monocyte chemotactic protein (MCP)-1, macrophage inflammatory protein (MIP)-1α, IL-6/IL-10, and receptor for advanced glycation end products in plasma and in IFN-γ, IL-6, IL-8, IL-10, MCP-1, MIP-1α, MIP-1β, IL-8/IL-10, and TNF-α in CSF.

**Conclusions:**

Substantial pro- and anti-inflammatory activity in the central neural system after surgery was found. If confirmed by larger studies, persistent changes in cytokine levels may serve as biomarkers for novel clinical trials.

**Electronic supplementary material:**

The online version of this article (doi:10.1186/s12974-016-0681-9) contains supplementary material, which is available to authorized users.

## Background

Cognitive impairment, including the conditions of postoperative delirium [1, 2] and postoperative cognitive dysfunction (POCD) [2, 3], is frequently observed after major surgery, particularly in older patients [3–7]. Postoperative delirium is associated with increases in length of hospital stay, admissions to long-term care institutions, long-term functional decline, and long-term mortality [8]. POCD, which is measured by cognitive testing, has been similarly shown to be associated with premature inability to work, dependency on social transfer payments, inability to cope independently, and long-term mortality [7, 9]. Although risk factors such as advanced age and neurodegenerative processes have been identified [5], the pathophysiology for either condition is still unclear.

Orthopedic procedures may result in extensive trauma, tissue injury, and blood loss, which can engage the innate immune system [10]. Preclinical studies suggest that inflammation is a possible pathogenic mechanism for POCD [11–15]. In particular, increased expression of cytokines in a rodent’s hippocampus following surgery was associated with cognitive decline [12, 16, 17]. Putative mediators of this postoperative neuroinflammatory response include interleukin (IL)-1β [12], IL-6, tumor necrosis factor-α (TNF-α), and high-mobility group box 1 protein (HMGB1) [16, 18].

Some studies suggest that surgical patients also exhibit elevations of proinflammatory cytokines in the central nervous system and systemic circulation [19–23]. However, three of these studies were performed in patients who had sustained traumatic injury prior to surgery; thus, the preceding trauma may have contributed to inflammation [19–21]. One study was performed in patients who underwent correction of idiopathic nasal cerebrospinal fluid (CSF) leak [24]. In this setting, the perforation of the blood-brain barrier due to the underlying pathology may have led to biomarker increases in the CSF that cannot be clearly differentiated from the changes induced by surgery. Another research team evaluated biomarkers in patients who received concurrent continuous infusion of bupivacaine into the spinal space through the same indwelling spinal catheter that was used for sampling [22, 23, 25, 26]. Given bupivacaine’s anti-inflammatory properties and potential association with chemical meningitis [27, 28], the reported results may have been confounded by this concurrent administration [29–31].

To address the potential relationship between surgery, systemic and central inflammatory biomarkers, and postoperative cognitive changes, we designed a prospective cohort pilot study. The study was performed in patients undergoing elective major knee surgery, a clinical equivalent of our previous preclinical model of aseptic surgery [11–13].

We hypothesized that aseptic surgery resulted in systemic inflammation and inflammation of the central nervous system. Results from this study will enable us to design future larger studies to test the hypothesis that persistent and excessive neuroinflammation caused by aseptic surgery triggers postoperative cognitive changes.

## Methods

### Study population

We included patients aged ≥55 years that were scheduled for elective major knee surgery who spoke English as the primary language. We included a broader age range to maximize patient recruitment. Exclusion criteria were pre-existing impaired cognition and contraindications for spinal anesthesia including anticoagulants, recent history of alcohol or drug abuse, or opioid tolerance.

The preoperative interview was conducted by a trained research assistant. Information from the preoperative physical exam including American Society of Anesthesiologists classification, preoperative use of opioids and benzodiazepines, and preoperative pain level (using the eleven-point numeric rating scales (NRS) from “no pain” (0/10) to “worst possible pain” (10/10) [32]) were recorded.

### Cognitive testing

All cognitive testing was performed by research assistants who had received training in the administration of these tests and were supervised by an experienced investigator (JL) [33]. In addition, all cognitive results were validated by a third investigator with advanced training in cognitive testing (LS). Delirium was determined using the confusion assessment method (CAM) [34] pre- and postoperatively daily until hospital discharge. CAM was developed as a screening instrument based on operationalization of the Diagnostic and Statistical Manual of Mental Disorders Version III, Revised (DSM-III-R) criteria for use by clinicians not formally trained in psychiatry in high-risk settings. Identifying delirium using CAM requires the presence of acute onset and fluctuating course, inattention, and either disorganized thinking and/or altered level of consciousness. If administered by trained research personnel to patients with sufficient level of arousal to be assessed, CAM has a sensitivity of 94–100 %, a specificity of 90–95 %, a high inter-observer reliability [34] and a convergent agreement with four other cognitive status tests. The research assistants were trained in the use of CAM until the inter-rater reliability reaches 0.96, based on a detailed manual developed for the administration of CAM [34]. At approximately 24 h after surgery, the patient was rated on the Richmond Agitation and Sedation Scale (RASS) [35]. If a patient was too sedated to be interviewed (RASS score of −4 or −5), delirium status could not be assessed.

POCD was determined using a battery of validated cognitive tests preoperatively and on postoperative days 1 to 3. The word list test measures the patients’ verbal memory [36]. The verbal fluency measures executive function and linguistic skills. The digit symbol test measures incidental memory, scanning, and motor speed [37]. Perioperative changes in scores for the cognitive tests were computed separately for each test by subtracting the postoperative test scores from the preoperative test score. Prior research validated that decline of four or more points on the word list or a decline of seven in the symbol or verbal fluency represents significant decline in cognitive performance [36–38]. Patients without delirium who decline on at least two cognitive tests for any postoperative day were designated as having POCD [38].

### Study protocol

A femoral nerve catheter was placed immediately preoperatively. In the operating room and after intravenous administration of 1–2 mg of midazolam to reduce anxiety, a spinal catheter was placed and baseline blood and CSF samples were collected. Subsequently, a single dose of hyperbaric bupivacaine 0.75 % was administered through the catheter. Intraoperatively, patients received intravenous propofol infusion titrated by the anesthesiologist for comfort while maintaining arousability and airway protection (Ramsay scores 4–5). Subsequent sampling of the blood and CSF was performed 3, 6, and 18 h after the end of the surgical procedure.

### Biochemical analysis

CSF and plasma samples were collected using appropriate tubes (Becton Dickinson Vacutainer, BD Diagnostics, NJ). Samples were immediately centrifuged at 1000 × *g* force for 20 min. and aliquots were stored at −80 °C.

### Cytokine testing

Matching plasma and CSF were assayed using a high-sensitivity Milliplex kit (Millipore, Billerica, MA) with antibody-coated beads for detection of interferon (IFN)-γ, IL-10, IL-12p70, IL-2, IL-4, IL-5, IL-6, IL-8, and TNF-α (standard curve range 0.13 to 2000 pg/mL). A standard sensitivity Milliplex Map kit (Millipore) was used for IFN-γ2, monocyte chemotactic protein (MCP)-1, macrophage inflammatory protein (MIP)-1α, and MIP-1β (standard curve range 3.2 to 10,000 pg/mL). A neurodegenerative Milliplex Map kit (Millipore) was used to measure amyloid β-40, amyloid β-42, and receptor for advanced glycation end products (RAGE) (standard curve range 3.4–15,000 pg/mL). Calprotectin (MRP8/14) was measured by enzyme-linked immunosorbent assay (ELISA) with a 1:40 dilution (BioLegend, San Diego; standard curve range 120 to 80,000 ng/mL). Testing was performed following the manufacturer’s protocols. Standard and patient samples were tested in duplicates. Milliplex results were acquired on a LABScan 200 analyzer (Luminex, Austin, TX) using Bio-Plex manager software (Bio-Rad, Hercules, CA), and study plates were compiled using Data Pro (Bio-Rad). ELISA assays were read by SoftMax Pro version 5. All multiplex analyses were done in the research laboratories of the Blood Centers of the Pacific, San Francisco, CA. Plasma and CSF IL-1β and HMGB1 were measured in duplicates using ELISA following the manufacturer’s protocols (R&D Systems, Minneapolis, MN; IBL International, Toronto, Ontario, Canada, and BioLegend, San Diego, CA, respectively).The detection limits for IL-1β as supplied by the manufacturer was 3.9–250 pg/mL and for HMGB1, 2.5–80 ng/mL (high-sensitivity assay procedure: 0.2–10 ng/mL; high sensitivity was used where regular assay procedure did not give a result).

### Statistical analysis

Inflammatory marker data are presented as mean (±standard deviation (SD)) and ranges in Additional file 1. Data analysis was performed using Microsoft® Excel and Statistics and Analytics System (SAS) V.9.3 for Windows. The linear mixed effects model was applied to statistically compare longitudinal changes (i.e., 3, 6, and 18 h) after surgery. To correct for multiple comparisons within a type of cytokine, we first conducted an overall test for change and then computed post hoc tests only when the overall test was significant. For the other research question about whether change occurred for any of the 17 assessments of plasma levels of cytokines and 15 assessments of CSF levels of cytokines, a correction for multiple comparisons would be necessary (data not shown).

### Definitions

#### Dependent variables

Separate linear mixed effects models were fitted for different plasma-CSF cytokines. The dependent variables for a single model are differences between the preoperative measure and the postoperatively measured cytokine level at 3, 6, and 18 h, respectively, of a specific inflammatory marker. The linear mixed effects model takes into account within subject correlations among the changes in the cytokine from the baseline over time. Between-subject changes in cytokines over time were treated as independent.

#### Independent variables

The primary independent variable group is defined as the presence or absence of POCD or delirium. This allowed assessment of whether there were different trends in the changes of postoperative cytokine from the preoperative measurements between subjects with and without POCD/delirium. The independent variables or covariates that were not significant (*p* value > 0.1) were excluded from the model, except for the case when no independent variable is significant; in this case, the group effect is the only included independent variable. Included in each model was a time variable to determine whether there were changes in cytokine levels across the three repeated measurements (3, 6, and 18 h after surgery). In addition, we incorporated a group*time interaction to test whether the two groups differed in patterns of change over time.

#### Statistical approach

To test the hypothesis (cytokine at *x* hours significantly changed from the preoperative measurement) is equivalent to test whether a linear combination of the fixed effects coefficients is different from 0. For example, if the fixed effects contain linear time trend, quadratic time trend, and the group effect, the mean of change in cytokine at time *t* from the baseline is ${\beta}_0+{\beta}_1t+{\beta}_2{t}^2+\mathrm{group}\;1$ for the subjects with POCD/delirium and ${\beta}_0+{\beta}_1t+{\beta}_2{t}^2+\mathrm{group}\;0$ for the subjects without POCD/delirium, respectively. Thus, to test the hypothesis that the change for group 1 (i.e., the POCD/delirium group) at time *t* is not 0 is equivalent to test ${\beta}_0+{\beta}_1t+{\beta}_2{t}^2+\mathrm{group}\;1\ne 0$, which can be achieved using the “contrast” or “estimate” functions in SAS.

Determination of the covariance structure (e.g., either random intercept or random intercept and slope model was applied) was based on the Akaike information criterion (AIC). The model parameters were estimated by the restricted maximum likelihood method. The standard errors were computed using the Kackar and Harville approximation [39], and degrees of freedom were computed by the Kenward and Rogers [40] method.

## Results

Recruitment is summarized in a summary and flow diagram in Additional file 2. A total of 141 patients were asked to participate in the study between 12/2010 and 4/2013. Sixty-nine patients declined, 29 patients were ineligible due to medical conditions, and 17 for other reasons. Eight patients were removed after starting the study (causes explained in Additional file 2). Sampling was performed without complications in ten patients; this cohort forms the basis of the report. Nine patients underwent total knee arthroplasty, and one patient had a knee extensor tendon reconstruction with allograft. The mean age of the patients was 70.3 (8.7) years, range 59–84 years. The average blood loss was 105 (16) ml, average tourniquet time was 21 (21) min, and bone cement was used in 9/10 patients. At 24 h after surgery, the catheter was removed in all patients. No patient developed complications related to the study procedure. CSF and plasma cytokine analysis was performed in all ten patients. Demographics and comorbidities are summarized in Table 1 (patient characteristics). Data for the patients that underwent baseline cognitive testing, but were ultimately not included in the study, are provided in Additional file 3c.

**Table 1:** Patient demographics. A trained research assistant assessed health and demographic information associated with postoperative delirium, including age, gender, race, level of education, daily alcohol consumption, and baseline cognitive status. Patients were as well evaluated for the presence of CNS disorders (history of stroke or transient ischemic attacks, delirium, dementia, depression, and seizure) by chart review and through the perioperative interview. Patient 006 is the patient that developed delirium

	Gender	Age	History of CNS disorders	Other comorbidities	ASA
Patient-001	F	66		Depression	2
Patient-003	F	84		Hypertension	2
Patient-004	M	76		General Fatigue	2
Patient-005	M	75		Ischemic heart disease	2
Patient-006 (delirium)	M	75		Hypertension, valvular heart disease	2
Patient-007	M	61		Ischemic heart disease, hypertension, valvular heart disease, arrhythmia, COPD, melanoma	3
Patient-008	F	64	Psychiatric	Depression, hypertension, asthma, colon and endometrial cancer	2
Patient-009	M	80		Hypertension, renal disease	2
Patient-010	M	63		Hypertension, diabetes	2
Patient-011	M	59		Asthma	1

### Postoperative delirium

Preoperatively and on the subsequent two postoperative days, CAM could be performed on all patients as none were too sedated (Additional file 3a). For eight of the patients, the test for delirium was obtained on postoperative day 3 as well, since two patients were discharged after 2 days.

One patient developed postoperative delirium on day 1 which was resolved and was not detected in the subsequent two postoperative days of observation.

#### Postoperative cognitive dysfunction

Four patients (patient numbers 4, 5, 8, and 10) had evidence of a significant decline in two tests for POCD on post operative day 1, two on postoperative day 2 (patients 4 and 5), and four on postoperative day 3 (patients 3, 4, 5, and 9). Of note, four patients did not have cognitive test results for POCD on postoperative day 3. Patient 5 had a decline in all three tests on postoperative day 2. The detailed tests and interpretation of results are summarized in Additional file 3b. In none of the patients that tested positively for POCD the cognitive impairment was noticed by caregivers and study personnel not involved in cognitive testing or commented on by the patients during visits.

#### Inflammatory response after surgery in patients with and without postoperative cognitive changes

Biochemical analyses were performed in ten patients with complete sample sets. Due to the sample size, patients with postoperative cognitive changes (delirium and POCD) were statistically analyzed in one group.

**Fig. 1:**
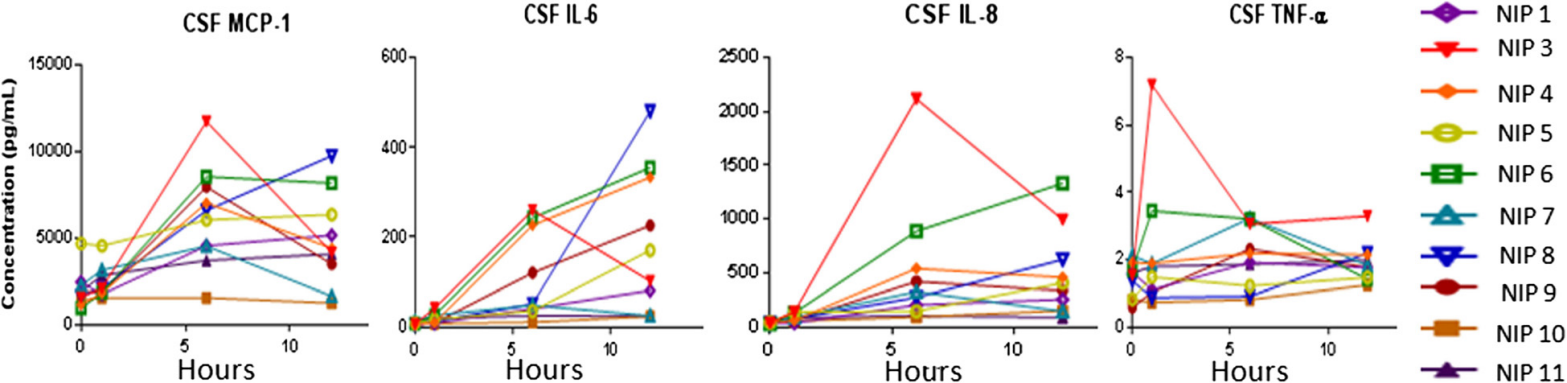
Time course of inflammatory markers MCP-1, interleukins 6 and 8, and tumor necrosis factor α over the perioperative period shows increased CSF levels in some patients. Of these, NIP 6 is the patient who developed postoperative delirium, NIP 3, 5, 8, and 9 developed POCD. Conversely, NIP 4 and 10 had no increase in these cytokines while developing POCD

**Fig. 2:**
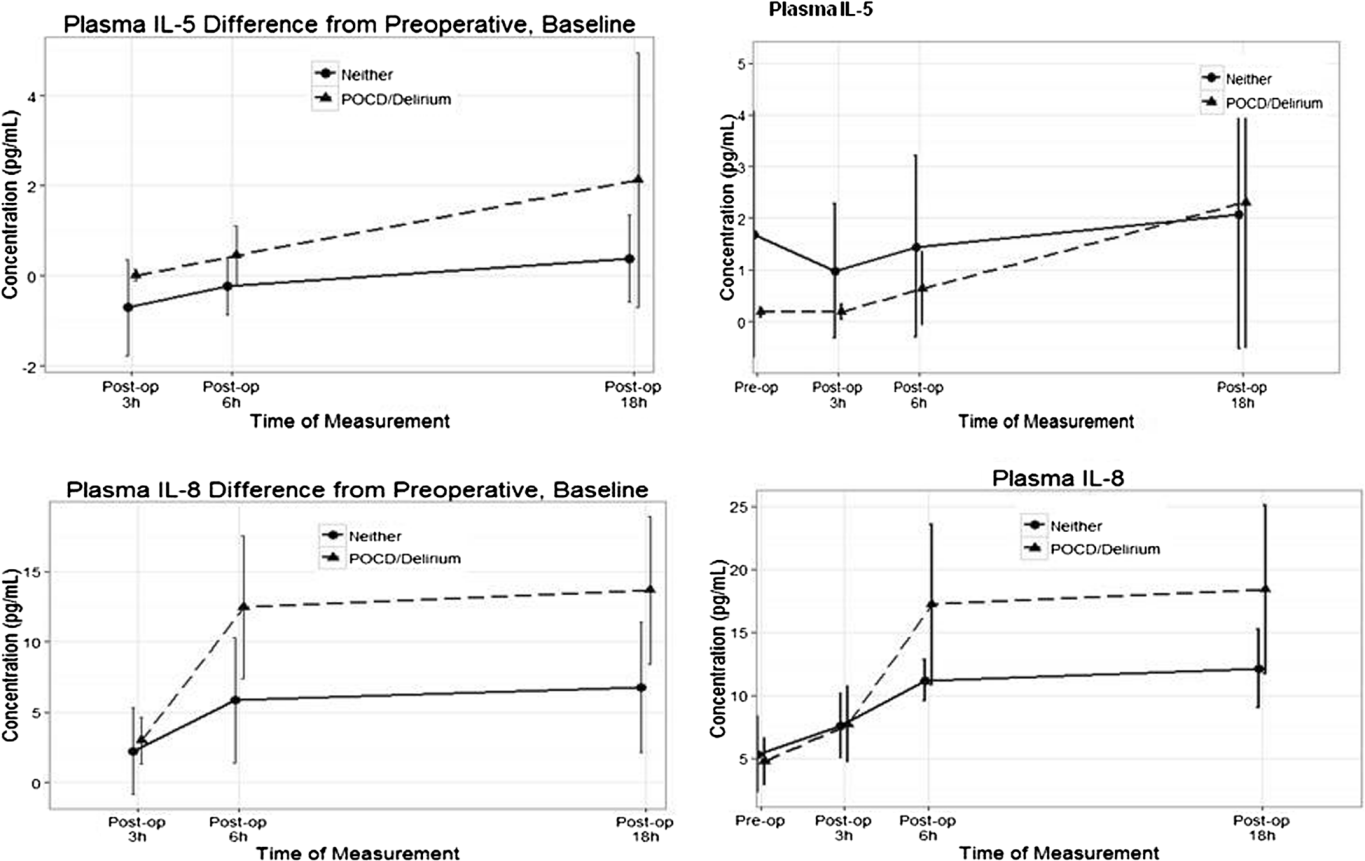
*Left Column:* Calculated difference between postoperative measurements and baseline measurements for plasma IL-5 and IL-8, plotted separately for patients with and without postoperative delirium and POCD. There was a significant difference in the sub-analyses at baseline vs. 18 h for IL-5 in the subjects with postoperative delirium or POCD and baseline vs. 6 and 18 h for IL-8 (see Table 2) in both groups. *Right Column:* The measured concentrations for both cytokines, as well plotted separately, for patients with and without POCD/delirium

We observed an increase in proinflammatory cytokines from the baseline, in particular, in CSF, in the first 18 h after surgery (Figs. 1 and 2, Tables 2 and 3). Data and descriptive statistics are provided in Additional file 1 and detailed statistical results on all parameters in Additional file 4.

**Table 2:** Standardized estimates and *p* values of the group effects and the contrasts for plasma. This table provides the standardized estimates and *p* values for the group effects (POCD/delirium (DLR) or no POCD/DLR) and the contrasts for plasma in the linear mixed effects model. The time*group interaction is not significant for any of the markers. Assessment of change at each postoperative time point was only for the plasma cytokines that have a (marginally) significant overall time trend. The contrasts “change from the preoperative measure to 3 h,” “change from the preoperative measure to 6 h,” and “change from the preoperative measure to 18 h” signify whether there was a significant change in the concentration of a specific marker at 3, 6, and 18 h after surgery compared to the preoperative measurement

Marker (plasma)	Change from the pre-op to 3 h	Change from the pre-op to 6 h	Change from the pre-op to 18 h	POCD/delirium*
Plasma amyloid β-42	−0.4978	−1.9768*	0.1621	–
Plasma IL-5				
Group 1	0.3460	1.8097	2.4810**	2.0170*
Group 0	−2.1696*	−1.2485	1.3206	
Plasma IL-6	0.6607	5.0300***	7.5368***	–
Plasma IL-8				
Group 1	2.7407**	7.0220***	7.6799***	2.0873*
Group 0	−0.0862	3.5706***	4.1325***	
Plasma IL-10	1.3315	2.9038***	5.1079***	–
Plasma MCP-1	1.8755*	4.3376***	1.8720*	–
Plasma IL-6/IL-10	0.7498	1.6455	3.5501***	
Plasma MIP-1α	2.9280**	2.7353**	1.2621	–

The standardized estimates were calculated by dividing the estimated values by the corresponding standard errors to achieve the same scale. The asterisks indicate the level of significance for the hypothesis that the group effect/contrast is different from zero. For plasma IL-5 and plasma IL-8, the comparisons between postoperative measures to the preoperative measure are displayed separately for the two groups (group 1 = POCD/DLR, group 0 = no POCD/DLR), since the groups’ main effect is marginally significant for these two markers. Of note, the time*group interaction is still not significant. All values are in pg ml^−1^. See Fig. 2 for explanation of the group effect on the differences between the preoperative (pre-op) measure and each postoperative measure

*0.05 < *p* value < 0.1; **0.01 < *p* value < 0.05; ***p value < 0.01

**Table 3:** Standardized estimates and *p* values of the group effects and the contrasts for CSF. This table provides the standardized estimates and *p* values for the group effects (POCD/delirium (DLR) or no POCD/DLR) and the contrasts for CSF in the linear mixed effects model. The statistical significance of change at each postoperative time point was tested as a contrast only for the CSF cytokines that have a (marginally) significant overall time trend in the changes at postoperative time points from the preoperative measure. For these markers, neither of the group effect nor the time*group interaction was significant, and therefore, they were excluded from the models. The contrasts “change from the preoperative measure to 3 h,” “change from the preoperative measure to 6 h,” and “change from the preoperative measure to 18 h” signify whether there was a significant change in the concentration of a specific cytokine at 3, 6, and 18 h after surgery compared to the preoperative measurement. All values are in pg ml^−1^

Marker (CSF)	Change from the pre-op to 3 h	Change from the pre-op to 6 h	Change from the pre-op to 18 h	POCD/delirium
CSF IL-5	−2.0244*	0.7591	2.1854**	–
CSF IL-6	1.2427	2.5724**	5.1566***	–
CSF IL-8	0.3551	3.4991***	3.2646***	–
CSF IL-12p70	−0.0414	1.8853*	0.9397	–
CSF MCP-1	0.4484	5.3716***	3.6158***	–
CSF MIP-1α	3.0862**	3.3400***	4.6466***	–
CSF MIP-1β	3.9098***	4.0916***	2.8341**	–
CSF IL-8/IL-10	0.2125	−1.8157*	−1.4464	

*0.05 < *p* value < 0.1; **0.01 < *p* value < 0.05; ***p value < 0.01

Plasma IL-5 and plasma IL-8 showed a strong different trend of their values from the preoperative measure between patients with and without postoperative cognitive changes (Table 2 and Fig. 2). While the overall difference between groups was not statistically significant (*p* = 0.0784 and *p* = 0.0703, respectively), there were statistically significant differences between preoperative and postoperative values at different time points. For IL-5, there was a statistically significant increase in the POCD/delirium group at 18 h after surgery compared to baseline (*p* = 0.0333) (Fig. 2 and Table 2). For IL-8, we identified a significant increase in the POCD/delirium group compared to baseline at all time points (3 h *p* = 0.0161; 6 h *p* < 0.0001; 18 h *p* < 0.0001), and in the group without cognitive postoperative changes at 6 h (*p* = 0.0039) and 18 h (*p* = 0.0014).

Some of the other plasma cytokines had significantly elevated postoperative values with a (marginally) significant time trend in the postoperative changes but without a clear trend between patients with and without postoperative cognitive changes (Table 2): IL-6 (3 h *p* = 0.5151; 6 h *p* < 0.0001; 18 h *p* < 0.0001), MCP-1 (3 h *p* = 0.0821; 6 h *p* = 0.0007; 18 h *p* = 0.0826), IL-6/IL-10 (3 h *p* = 0.4634; 6 h *p* = 0.1278; 18 h *p* = 0.0016), and MIP-1α (3 h *p* = 0.0132; 6 h *p* = 0.0217; 18 h *p* = 0.2268), as well as of the anti-inflammatory IL-10 (3 h *p* = 0.1938; 6 h *p* = 0.0071; 18 h *p* < 0.0001).

We observed that there was a (marginally) significant time trend for CSF markers but without a clear difference between the two groups. A (marginally) significant change was found for proinflammatory IL-5 (3 h *p* = 0.0626; 6 h *p* = 0.4604; 18 h *p* = 0.0465), IL-6 (3 h *p* = 0.2297; 6 h *p* = 0.0254; 18 h *p* < 0.0001), IL-8 (3 h *p* = 0.7260; 6 h *p* = 0.0021; 18 h *p* = 0.0037), MCP-1 (3 h *p* = 0.6580; 6 h *p* < 0.0001; 18 h *p* = 0.0014), MIP-1α (3 h *p* = 0.0130; 6 h *p* = 0.0087; 18 h *p* = 0.0012), MIP-1β (3 h *p* = 0.0036; 6 h *p* = 0.0027; 18 h *p* = 0.0196) (Table 3), and IL-8/IL-10 (3 h *p* = 0.8340; 6 h *p* = 0.0858; 18 h *p* = 0.1649). Figure 1 illustrates the CSF course of the four proinflammatory cytokines MCP-1, IL-6, IL-8, and TNF-α for all patients.

IL-1β was below the detection threshold for most samples in both plasma and CSF. For HMGB1, we had previously reported a similar pattern with substantial increases in a smaller subset of these patients [18].

For the plasma and CSF cytokines that did not have a significant overall time trend in the postoperative changes from the preoperative measure, the significance of the mean postoperative changes was tested. For each cytokine, the linear mixed effects model was applied only including the group effect (i.e., POCD/delirium or no POCD/delirium). Given that the group effect was not significant, we tested if the mean change between the postoperative and the preoperative level is significantly different from 0 considering all the postoperative time points and the two groups together. The correlation among observations of the same subject is accounted for by the random intercept model.

In the *plasma*, there was a significant increase after surgery in the mean level of RAGE (*p* = 0.0064) (see Additional file 4) and also a marginally significant decrease in IL-12p70 (*p* = 0.0735) were found by testing the fixed intercept term of the linear mixed model. In the *CSF*, a significant increase in the mean level of IL-10 (*p* = 0.0421) and IFN-γ (*p* = 0.0420) and also a marginally significant increase in TNF-α (*p* = 0.0885) were detected.

#### Cytokine profile in the patient who developed postoperative delirium

In the patient with postoperative delirium, several plasma cytokines were elevated at baseline relatively to the other patients: calprotectin (5-fold elevation), MIP-1α (2-fold), MIP-1β (3-fold), and IL-6 (14-fold) (see Additional files 1 and 4); these cytokines remained elevated at the later time points. Baseline and postoperative values for several cytokines were relatively lower than in the other patients: IFN-α2 (4-fold decrease), IFN-γ (>20-fold), IL-4 (10-fold), IL-5 (20-fold), IL-12(p70) (>20-fold), and amyloid β-40 (5-fold). In CSF, baseline values for calprotectin were relatively increased (1.5-fold), while others were decreased: IFN-α2 (8-fold) and RAGE (20-fold). Several CSF cytokines increased more pronouncedly over preoperative values than in patients without delirium: IL-6 (2-fold), IL-8 (2–4-fold), and calprotectin (2–25-fold). The increase of CSF IL-6, IL-8, and MCP-1 persisted longer in this patient. RAGE was initially 15-fold decreased with a relatively more pronounced increase than in other patients, while early anti-inflammatory IL-10 values were relatively (20-fold) smaller.

#### Cytokine profile in patients who developed POCD

Baseline values for several plasma cytokines were relatively lower in patients that later developed to POCD: IL-4 (10-fold lower), IL-5 (8-fold), IL-6 (8-fold), IL-12(p-70) (20-fold), IFN-α2 (8-fold), and IFN-γ (4-fold). While the other cytokines approximated preoperative values for the patients with POCD postoperatively, several plasma cytokine levels remained relatively lower in these patients: IFN-α2 (3–20-fold), IL-12(p70) (>20-fold), and IL-4 (5–20-fold). In CSF, some cytokines were relatively higher at baseline: IFN-α2 (6-fold), amyloid β-40 (2-fold), and IL-10 (3-fold). IL-10 values remained relatively elevated throughout the observation period (3-fold) in patients who developed POCD.

#### Potential markers to identify patients at risk for delirium and POCD prior to surgery

Preoperatively, plasma IL-6 was 14-fold higher at baseline in the one patient who developed delirium; for calprotectin, more than 5-fold higher values, and for plasma, MIP-1β more than 3-fold higher values were observed. Conversely, plasma IFN-α2 was 4-fold lower, IL-4 was 10-fold lower, IL-5 was 20-fold lower, IFN-γ and IL-12(p70) were more than 20-fold lower, Aβ-40 and Aβ-42 were more than 5-fold lower in this patient. In CSF, IFN-α2 and RAGE baseline levels were more than 10-fold lower in the patient with postoperative delirium.

Patients who developed POCD had 3-fold increased in baseline levels of plasma IL-10, 2-fold increased in baseline of CSF Aβ-40, and 6-fold increased in baseline levels of CSF IFN-α. Baseline values for plasma IFN-α2, IL-4, IL-5, and IL-6 were decreased 8-fold, and IL-12(p70) more than 10-fold.

#### Potential plasma markers that reflect central nervous system inflammatory changes

Cytokines with significant plasma-CSF correlations were IL-6 (*r* = 0.47), IL-8 (*r* = 0.51), MIP-1α (*r* = 0.70), and MIP-1β (*r* = 0.43). Correlations of other cytokines between plasma and CSF values were not significant.

## Discussion

We observed substantial elevations in proinflammatory cytokines in both plasma and CSF after aseptic surgery (Tables 2 and 3). The pronounced changes in CSF cytokines compared to plasma for several cytokines (MCP, MIP-1α, MIP-1β) provide evidence for substantial inflammatory activity in the central nervous system. In particular, the statistically significant increases in plasma IL-5, IL-6, IL-8, MCP-1, MIP-1α, and RAGE and of anti-inflammatory IL-10 suggest a substantial activation of key pathways of the immune response. The activation of CSF IL-5, IL-6, IL-8, MCP-1, MIP-1α, and MIP-1β indicates that chemoattraction of monocytes may play a key role similar to what had been previously observed in preclinical studies [14, 18, 41]. Moreover, the cytokine pattern changes suggest an immunological response that includes B-cell stimulation, immunoglobulin secretion, activation of T-cells, eosinophil and neutrophil granulocytes, and simultaneous activation of anti-inflammatory pathways. Release of mitochondrial damage-associated molecular patterns from femur fracture reamings also activate neutrophils, which release IL-8 and MMP9 that contribute to remote organ injury [42, 43].

Due to its sample size, this was a feasibility study with predominantly descriptive findings. Our results cannot determine any causal association of cytokine changes with postoperative cognitive changes. However, there were marginally significant differences in the postoperative changes of plasma IL-5 and IL-8 from baseline in patients with and without postoperative cognitive changes (Fig. 2 and Table 2). This result provides a starting point for future research.

In addition, our preliminary results show that proinflammatory plasma cytokines were substantially elevated in the patient who developed delirium. Moreover, a persistent increase in proinflammatory cytokines IL-6, IL-8, and MCP-1 was observed in the CSF of this patient. It is noteworthy that this patient, while meeting criteria for elective surgery, was among the older patients in our cohort and among these with more comorbidities. It remains to be proven whether preoperative subclinical inflammation may be a predictor for postoperative delirium.

Patients who developed POCD had decreased levels of several anti- and proinflammatory cytokines. Equally, our findings for POCD are limited by the size of the dataset and the relatively discrete signs of POCD that were observed in 60 % of the patients. Further studies are needed to confirm these alterations.

Based on our results, an important consideration for future work may be the altered levels of several cytokines at baseline in patients who later developed POCD or delirium. Currently, the significance of these alterations is unclear and needs to be investigated in future studies with larger sample size. Research to further elucidate these alterations may be useful to identify patients who are at risk for postoperative surgical changes. However, while significant correlations between plasma and CSF samples were present for IL-6, IL-8, MIP-1α, and MIP-1β, correlations were negligible for other cytokines. This is a potentially important result of our work, since it may indicate limits to the utility of measurements of some cytokines in more readily available plasma samples, and the potentially usefulness of this approach for others. Our results support the conclusions from a recent meta-analysis [44] that found a correlation between POCD and the peripheral markers of IL-6 and S100β [44] but indicate limited applicability of plasma approaches for other cytokines.

The scientific context of our results contains animal studies that demonstrate that surgical stimulus activates the immune system even under aseptic conditions [10]. Several preclinical studies have established inflammation of the central nervous system as a pathogenic mechanism for cognitive dysfunction [12–15, 45]. Release of proinflammatory mediators including damage-associated molecular patterns (DAMPs), such as HMGB1, into the circulation have been shown to impair the blood-brain barrier permeability after orthopedic surgery in mice [14]. These cytokines converge into activation of the innate immune system by convergence on NF-кB pathways. In the circulation, HMGB1 interacts with pattern recognition receptors (toll-like receptors 2 and 4 as well as receptor for advanced glycation end products) on immunocytes. Through a permeable blood-brain barrier, peripheral monocyte-derived macrophages access the brain parenchyma. These are attracted by signaling from the hippocampal MCP-1, a chemokine that regulates migration and infiltration of C–C motif receptor 2-expressing cells [41, 46, 47]. These processes have been associated with memory dysfunction in preclinical models of POCD, and strategies to block macrophage infiltration and/or excessive proinflammatory mediators have been successful in reducing these memory deficits [14, 48]. Macrophage-specific IкB kinase (IKK)β coordinates activation of NF-кB; when it is deleted, it prevents the blood-brain barrier disruption and infiltration of bone marrow-derived macrophages into the hippocampus following surgery [48]. Learning and memory processes rely on the hippocampus. Rodent studies have shown that increased expression of cytokines and DAMPs in rodent’s hippocampus following surgery was associated with cognitive decline [12, 16, 17]. Potential mediators identified in animal models include IL-1β [12], IL-6, TNF-α, and HMGB1 [16, 18]. Existing clinical studies show accumulating evidence that the CSF’s isolation from inflammatory reactions (immune privilege) [49] may not be sustained perioperatively. Baune et al. [50] reported a correlation between plasma IL-8 levels and cognitive performance in 369 community-dwelling elderly subjects. Beloosesky and coworkers [19] investigated the functional status and plasma cytokine levels in 41 hip fracture patients. They reported significantly higher plasma C-reactive protein and IL-6 kinetics curves in patients with preoperatively impaired mental status. Recent studies in patients undergoing emergency surgery reported that preoperative inflammatory markers were elevated in plasma and CSF [20] [21] and that the extent of this inflammatory reaction might correlate with the degree of postoperative cognitive decline [20]. In these studies, the cause of the observed biomarker increase cannot be clearly attributed to surgery.

The change in CSF/plasma albumin ratio after aseptic peripheral surgery [22] indicates an alteration in the blood-brain barrier permeability in the perioperative period. When evaluating plasma and CSF samples for up to 30 h postoperatively, a consistent upregulation of CSF IL-6, a transient increase in CSF IL-8, a delayed increase in PGE2, and undetectable IL-1β, TNF-α, and IL-10 in CSF [25] were found.

Several investigations have been performed in one patient population [22, 23, 25, 26]. Bromander et al. [23] investigated changes in CSF and serum in 35 patients undergoing knee arthroplasty surgery preoperatively, 3 and 24 h postoperatively. It was determined that IL-2, IL-8, IL-10, and IL-13 were increased to more than 500 % of their initial concentrations, while TNF content doubled [23]. However, the concurrent administration of bupivacaine in these studies may have affected the reported results. Bupivacaine has known anti-inflammatory properties [29–31, 51] and has been associated with chemical meningitis [27, 28]. Of note, patients in our study group received a single dose of bupivacaine at the time of placement of the catheter with no subsequent administrations.

Tang [24] measured cytokines in 11 patients undergoing endoscopic surgery to correct idiopathic nasal CSF drip under general anesthesia. Mean CSF Aβ-42 remained unchanged, but total-tau and phosphorylated-tau181P increased progressively until 48 h after surgery. CSF IL-10, S100β, IL-6, and TNF-α were increased at 24 h. Their results may indicate neuronal damage and/or aggravation of existing conditions. However, the underlying CSF rhinorrhea [24] implies a breach of the blood-brain barrier, potentially causing direct immune activation in the CSF, and meningitis is a potential complication [52]. In addition, the condition is frequently associated with intracranial hypertension [53], and fluorescein was injected via the lumbar catheter into the CSF during this surgery [54]. Cape and coworkers [55] assessed 43 patients aged >60 years with acute hip fracture for delirium before and 3–4 days after surgery. CSF samples were taken at the induction of spinal anesthesia. Delirium was diagnosed in eight patients before surgery; 17 patients developed delirium after surgery. The authors found that CSF IL-1β was significantly higher in patients with new-onset delirium compared to patients without delirium and that CSF to serum IL-1β ratios were higher in delirious than non-delirious patients. CSF IL-1ra was significantly higher in patients with delirium before surgery compared to new-onset delirium.

In the context of the above literature, our study is the first to demonstrate significant perioperative changes in a wide range of inflammatory markers in a setting without trauma and with minimal injury to the cerebrospinal space. Interestingly, these findings are more pronounced in CSF than in plasma, which could indicate that they originate from the central nervous system.

There are, however, several potential limitations to our study. We used CAM to determine the presence of postoperative delirium. CAM was developed as a screening instrument for delirium but has high sensitivity and specificity. When compared with the delirium rating scale, which has ten features, CAM measured 9/10 features except for physical disorder [56]. Whether using the delirium rating scale will yield different results will need to be evaluated by future studies. In addition, the follow-up period limits our data on POCD. Third, because of the small sample size, our study results will need validation by a larger study. We included a broader age range of patients in order to optimize patient recruitment. The inclusion of older subjects may result in a higher incidence of postoperative delirium or POCD. Additional considerations include more frequent CSF and plasma sampling over a more extended period to consider other than the effect of circadian variations [57]. Moreover, it is currently unknown to what extent intravenous sedation [58] and a single bolus injection of bupivacaine for spinal anesthesia affects cytokine levels in CSF and plasma. An inflammatory response as a result of the in-dwelling catheter cannot be excluded from our data. Preoperative medications, pain, and use of opioids and other drugs postoperatively may as well affect cognitive status. Lastly, idiosyncrasies of the surgical procedure [59, 60], such as tourniquet use [60, 61, 62] reaming of the femur [42, 43] with potential for fat emboli, and cementing of the prosthesis [63], may influence the results. Tourniquet times were 21.0 (20.7) min. Its role as a cofactor in this study is probably small, since cytokine patterns in the two patients with the longest tourniquet time (about 60 min) were not discernible from the remaining patients. Similarly, there was no difference in cytokine patterns in the patient that did not undergo reaming of the femur and cementing (data not shown).

## Conclusions

In summary, our results demonstrate increased inflammatory activity in plasma and CSF in the perioperative period and shed more light on the complex inflammatory mechanisms occurring during and after surgery. These results corroborate previous studies [23, 25] that a proinflammatory response based on IL-6 and IL-8 is present in CSF and, less markedly, in plasma after aseptic surgery. In addition, we found elevated levels of the HMGB1 receptor RAGE, consistent with previously reported elevated levels of HMGB1 [16, 18, 64] and TNF-α [13, 23] in both animal and clinical studies. Moreover, our results and previous work [23] establish a role for the anti-inflammatory cytokine IL-10. The role for IL-1β requires further clarification; while elevated levels were found in animal studies [12], we and others [25] were unable to reproduce this finding after aseptic surgery in humans. Further studies need to elucidate the crosstalk between systemic markers of inflammation and those present in the CSF. While we found significant alterations in some cytokines and substantial alterations in others in plasma and CSF from patients with postoperative cognitive changes, further research is needed to elucidate potential contributions to the pathogenesis of postoperative delirium and POCD.

## Additional files

Additional file 1: Cytokine data. (XLSX 73 kb) Additional file 2: Flow Chart describing study recruitment. (DOCX 39 kb) Additional file 3: Cognitive test, demographics and medication data. (DOCX 54 kb) Additional file 4: Cytokine detailed statistical results. (DOCX 104 kb)

## Abbreviations

Aβ: Amyloid beta
CAM: Confusion assessment method
CSF: Cerebrospinal fluid
DAMP: Damage-associated molecular pattern
DSM-III-R: Diagnostic and Statistical Manual of Mental Disorders Version III, Revised
ELISA: Enzyme-linked immunosorbent assay
HMGB: High-mobility group box (protein)
IFN: Interferon
IL: Interleukin
IRB: Institutional Review Board
MCP: Monocyte chemotactic protein
MIP: Macrophage inflammatory protein
MRP: Myeloid-related protein
NRS: Numeric rating scale
POCD: Postoperative cognitive dysfunction
RAGE: Receptor for advanced glycation end products
RASS: Richmond Agitation and Sedation Scale
SAS: Statistics and Analytics System
TNF: Tumor necrosis factor
