# Supplementary material for: Perioperative cerebrospinal fluid and plasma inflammatory markers after orthopedic surgery
Source: J Neuroinflammation. 2016 Aug 30;13(1):211. doi: 10.1186/s12974-016-0681-9 (PMC5006595; doi:10.1186/s12974-016-0681-9)
Supplement: Additional file 2: — Flow Chart describing study recruitment. (DOCX 39 kb) [file 12974_2016_681_MOESM2_ESM.docx]

# Additional file 1

A total of 141 patients were asked to participate in the study between 12/2010 and 4/2013. Sixty-nine patients declined, 29 patients were ineligible due to medical conditions (such as back pain or radiculopathy, high dose opioid therapy, need for anticoagulation therapy, etc.), 7 were excluded due to language barrier and 10 patients had the surgery cancelled or postponed. The remaining 26 patients were enrolled. Seven patients withdrew after the initial interview due to scheduling problems, 7 additional patients were removed after starting the study due to difficulties placing the spinal catheter and 1 patient refused continuation in the study after uneventful catheter placement and was removed from the study. In one additional patient, the catheter malfunctioned after surgery and no further sampling could be performed (NIP-2). Sampling was performed without complications in 10 patients; this cohort forms the basis of the report.
