# Supplementary material for: Perioperative cerebrospinal fluid and plasma inflammatory markers after orthopedic surgery
Source: J Neuroinflammation. 2016 Aug 30;13(1):211. doi: 10.1186/s12974-016-0681-9 (PMC5006595; doi:10.1186/s12974-016-0681-9)
Supplement: Additional file 3: — Cognitive test, demographics and medication data. (DOCX 54 kb) [file 12974_2016_681_MOESM3_ESM.docx]

**Cognitive test data**

- n=10, 1=CAM Positive, 9= CAM Negative
- NIP-006: CAM positive (highlighted in yellow)

## Supplement 1a: NIP CAM Results By Day

Table 1. POD 1 CAM

| **StudyNo** | **Acute Onset** | **Inattention** | **Fluctuating Inattention** | **Disorganized Thinking** | **Altered Level of Consciousness** | **Disorientation** | **Memory Impairment** | **Perceptual Disturbances** | **Hypermotor Activity** | **Hypormotor Activity** | **Altered Sleep-Wake Cycle** | **Feature 1** | **Feature 2** | **Feature 3** | **Feature 4** | **Delirium Diagnosis** | |  |
| --- | --- | --- | --- | --- | --- | --- | --- | --- | --- | --- | --- | --- | --- | --- | --- | --- | --- | --- |
| NIP-001 | 0 | 0 |  | 0 | 0 | 0 | 0 | 0 | 0 | 0 | 0 | 0 | 0 | 0 | 0 | | 0 | |
| NIP-003 | 0 | 0 |  | 0 | 0 | 0 | 0 | 0 | 0 | 0 | 0 | 0 | 0 | 0 | 0 | | 0 | |
| NIP-004 | 0 | 0 |  | 0 | 0 | 0 | 0 | 0 | 0 | 1 | 0 | 0 | 0 | 0 | 0 | | 0 | |
| NIP-005 | 0 | 0 |  | 0 | 0 | 0 | 0 | 0 | 0 | 0 | 0 | 0 | 0 | 0 | 0 | | 0 | |
| NIP-006 | 1 | 1 | 1 | 0 | 2 | 1 | 0 | 0 | 0 | 0 | 1 | 1 | 1 | 0 | 2 | | 1 | |
| NIP-007 | 1 | 0 |  | 0 | 2 | 0 | 0 | 0 | 0 | 0 | 0 | 1 | 0 | 0 | 2 | | 0 | |
| NIP-008 | 0 | 0 |  | 0 | 0 | 0 | 0 | 0 | 0 | 0 | 0 | 0 | 0 | 0 | 0 | | 0 | |
| NIP-009 | 0 | 0 |  | 0 | 0 | 0 | 0 | 0 | 0 | 0 | 0 | 0 | 0 | 0 | 0 | | 0 | |
| NIP-010 | 0 | 0 |  | 0 | 0 | 0 | 0 | 0 | 0 | 0 | 0 | 0 | 0 | 0 | 0 | | 0 | |
| NIP-011 | 0 | 0 |  | 0 | 0 | 0 | 0 | 0 | 0 | 0 | 0 | 0 | 0 | 0 | 0 | | 0 | |

Table 2. POD 2 CAM

| **StudyNo** | **Acute Onset** | **Inattention** | **Fluctuating Inattention** | **Disorganized Thinking** | **Altered Level of Consciousness** | **Disorientation** | **Memory Impairment** | **Perceptual Disturbances** | **Hypermotor Activity** | **Hypormotor Activity** | **Altered Sleep-Wake Cycle** | **Feature 1** | **Feature 2** | **Feature 3** | **Feature 4** | **Delirium Diagnosis** | |  |
| --- | --- | --- | --- | --- | --- | --- | --- | --- | --- | --- | --- | --- | --- | --- | --- | --- | --- | --- |
| NIP-001 | 0 | 0 |  | 0 | 0 | 0 | 0 | 0 | 0 | 0 | 0 | 0 | 0 | 0 | 0 | | 0 | |
| NIP-003 | 1 | 0 |  | 0 | 2 | 0 | 0 | 0 | 0 | 0 | 0 | 1 | 0 | 0 | 2 | | 0 | |
| NIP-004 | 0 | 0 |  | 0 | 0 | 0 | 0 | 0 | 0 | 1 | 0 | 0 | 0 | 0 | 0 | | 0 | |
| NIP-005 | 0 | 0 |  | 0 | 0 | 0 | 0 | 0 | 0 | 0 | 0 | 0 | 0 | 0 | 0 | | 0 | |
| NIP-006 | 0 | 0 |  | 0 | 0 | 0 | 0 | 0 | 0 | 0 | 0 | 0 | 0 | 0 | 0 | | 0 | |
| NIP-007 | 0 | 0 |  | 0 | 0 | 0 | 0 | 0 | 0 | 0 | 0 | 0 | 0 | 0 | 0 | | 0 | |
| NIP-008 | 0 | 0 |  | 0 | 0 | 0 | 0 | 0 | 0 | 0 | 0 | 0 | 0 | 0 | 0 | | 0 | |
| NIP-009 | 0 | 0 |  | 0 | 0 | 0 | 0 | 0 | 0 | 0 | 0 | 0 | 0 | 0 | 0 | | 0 | |
| NIP-010 | 0 | 0 |  | 0 | 0 | 0 | 0 | 0 | 0 | 0 | 0 | 0 | 0 | 0 | 0 | | 0 | |
| NIP-011 | 0 | 0 |  | 0 | 0 | 0 | 0 | 0 | 0 | 0 | 0 | 0 | 0 | 0 | 0 | | 0 | |

Table 3. POD 3 CAM

| **StudyNo** | **Acute Onset** | **Inattention** | **Fluctuating Inattention** | **Disorganized Thinking** | **Altered Level of Consciousness** | **Disorientation** | **Memory Impairment** | **Perceptual Disturbances** | **Hypermotor Activity** | **Hypormotor Activity** | **Altered Sleep-Wake Cycle** | **Feature 1** | **Feature 2** | **Feature 3** | **Feature 4** | **Delirium Diagnosis** |
| --- | --- | --- | --- | --- | --- | --- | --- | --- | --- | --- | --- | --- | --- | --- | --- | --- |
| NIP-001 | 0 | 0 |  | 0 | 0 | 0 | 0 | 0 | 0 | 0 | 0 | 0 | 0 | 0 | 0 | 0 |
| NIP-003 | 1 | 0 |  | 0 | 2 | 0 | 0 | 0 | 0 | 0 | 0 | 1 | 0 | 0 | 2 | 0 |
| NIP-004 | -- | -- |  | -- | -- | -- | -- | -- | -- | -- | -- | -- | -- | -- | -- | -- |
| NIP-005 | 0 | 0 |  | 0 | 0 | 0 | 0 | 0 | 0 | 0 | 0 | 0 | 0 | 0 | 0 | 0 |
| NIP-006 | 0 | 0 |  | 0 | 0 | 0 | 0 | 0 | 0 | 0 | 0 | 0 | 0 | 0 | 0 | 0 |
| NIP-007 | 0 | 0 |  | 0 | 0 | 0 | 0 | 0 | 0 | 0 | 0 | 0 | 0 | 0 | 0 | 0 |
| NIP-008 | -- | -- |  | -- | 0 | -- | -- | -- | -- | -- | -- | -- | -- | -- | -- | -- |
| NIP-009 | 0 | 0 |  | 0 | 0 | 0 | 0 | 0 | 0 | 0 | 0 | 0 | 0 | 0 | 0 | 0 |
| NIP-010 | 0 | 0 |  | 0 | 0 | 0 | 0 | 0 | 0 | 0 | 0 | 0 | 0 | 0 | 0 | 0 |
| NIP-011 | 0 | 0 |  | 0 | 0 | 0 | 0 | 0 | 0 | 0 | 0 | 0 | 0 | 0 | 0 | 0 |

## Supplement 2b Cognitive Dysfunction

***Definition of failure in a Cognitive task:***

- Digit Symbol (DS) test change 7 or more
- Verbal Fluency (VF) test 7 or more
- Word List (WL) test change 4 or more

***Definition of POCD on this postoperative day:***

- Failure in two or more tasks on this particular day

***Summary of positive test results for POCD:***

NIP 003:  declined on DS and VF on POD 3
NIP 004:  declined on DS and VF on POD 1-3
NIP 005:  declined on DS and VF on POD 1-3 and also on WL on POD 2
NIP 008:  declined on DS and VF on POD 1
NIP 009:  declined on DS and WL on POD 3
NIP 010:  declined on DS and VF on POD 1

***Detailed POCD test results:***

Table 1. Change from Preop Score for Digit Symbol by Day

| **StudyNo** | **POD1** | **POD2** | **POD3** |
| --- | --- | --- | --- |
| NIP-001 | -1.00 | +2.00 | +1.00 |
| NIP-003 | -6.00 | -7.00 | -11.00 |
| NIP-004 | -23.00 | -18.00 | no data |
| NIP-005 | -15.00 | -8.00 | -5.00 |
| NIP-006 | 0.00 | -2.00 | -2.00 |
| NIP-007 | 0.00 | -5.00 | -7.00 |
| NIP-008 | -12.00 | -9.00 | no data |
| NIP-009 | -4.00 | -4.00 | -10.00 |
| NIP-010 | -26.00 | -25.00 | no data |
| NIP-011 | -6.00 | -4.00 | no data |

Table 2. Total Score on Digit Symbol by day (Out of Possible 93)

| **StudyNo** | **Preop** | **POD1** | **POD2** | **POD3** |
| --- | --- | --- | --- | --- |
| NIP-001 | 26.00 | 25.00 | 28.00 | 27.00 |
| NIP-003 | 44.00 | 38.00 | 37.00 | 33.00 |
| NIP-004 | 46.00 | 23.00 | 28.00 | no data |
| NIP-005 | 41.00 | 26.00 | 33.00 | 36.00 |
| NIP-006 | 20.00 | 20.00 | 18.00 | 18.00 |
| NIP-007 | 30.00 | 30.00 | 25.00 | 23.00 |
| NIP-008 | 40.00 | 28.00 | 31.00 | no data |
| NIP-009 | 46.00 | 42.00 | 42.00 | 36.00 |
| NIP-010 | 55.00 | 29.00 | 30.00 | no data |
| NIP-011 | 59.00 | 53.00 | 55.00 | no data |

Table 3. Change from Preop Score for Verbal Fluency by Day

| **StudyNo** | **POD1** | **POD2** | **POD3** |
| --- | --- | --- | --- |
| NIP-001 | no data | no data | no data |
| NIP-003 | -3.00 | -5.00 | -11.00 |
| NIP-004 | -11.00 | -13.00 | no data |
| NIP-005 | -13.00 | -11.00 | -10.00 |
| NIP-006 | -3.00 | -5.00 | -4.00 |
| NIP-007 | +1.00 | 0.00 | -1.00 |
| NIP-008 | -13.00 | -4.00 | no data |
| NIP-009 | +6.00 | +10.00 | +12.00 |
| NIP-010 | -26.00 | -6.00 | no data |
| NIP-011 | +16.00 | +22.00 | no data |

Table 4. Total Score on both trails for Verbal Fluency by day (No Possible Maximum)

| **StudyNo** | **Preop** | **POD1** | **POD2** | **POD3** |
| --- | --- | --- | --- | --- |
| NIP-001 | no data | 5.00 | no data | 9.00 |
| NIP-003 | 17.00 | 14.00 | 12.00 | 6.00 |
| NIP-004 | 23.00 | 12.00 | 10.00 | 0.00 |
| NIP-005 | 29.00 | 16.00 | 18.00 | 19.00 |
| NIP-006 | 8.00 | 5.00 | 3.00 | 4.00 |
| NIP-007 | 16.00 | 17.00 | 16.00 | 15.00 |
| NIP-008 | 31.00 | 18.00 | 27.00 | 0.00 |
| NIP-009 | 14.00 | 20.00 | 24.00 | 26.00 |
| NIP-010 | 31.00 | 5.00 | 25.00 | 0.00 |
| NIP-011 | 20.00 | 36.00 | 42.00 | 0.00 |

Table 5. Change from Preop Score for Word List by Day

| **StudyNo** | **POD1** | **POD2** | **POD3** |
| --- | --- | --- | --- |
| NIP-001 | -2.00 | +2.00 | +2.00 |
| NIP-003 | 0.00 | -3.00 | -4.00 |
| NIP-004 | no data | -8.00 | no data |
| NIP-005 | -4.00 | -5.00 | -1.00 |
| NIP-006 | -10.00 | 0.00 | -1.00 |
| NIP-007 | -2.00 | 0.00 | -2.00 |
| NIP-008 | 0.00 | -1.00 | no data |
| NIP-009 | -3.00 | -1.00 | -7.00 |
| NIP-010 | 0.00 | +4.00 | no data |
| NIP-011 | -1.00 | -5.00 | no data |

Table 6. Total Score on 3 trials for Word List by Day

| \| **StudyNo** \| **Preop** \| **POD1** \| **POD2** \| **POD3** \| \| --- \| --- \| --- \| --- \| --- \| \| NIP-001 \| 21.00 \| 19.00 \| 23.00 \| 19.00 \| \| NIP-003 \| 21.00 \| 21.00 \| 18.00 \| 17.00 \| \| NIP-004 \| 19.00 \| no data \| 11.00 \| no data \| \| NIP-005 \| 16.00 \| 12.00 \| 21.00 \| 17.00 \| \| NIP-006 \| 10.00 \| 0.00 \| 10.00 \| 9.00 \| \| NIP-007 \| 18.00 \| 16.00 \| 18.00 \| 16.00 \| \| NIP-008 \| 18.00 \| 18.00 \| 17.00 \| no data \| \| NIP-009 \| 19.00 \| 16.00 \| 18.00 \| 12.00 \| \| NIP-010 \| 14.00 \| 14.00 \| 18.00 \| no data \| \| NIP-011 \| 20.00 \| 19.00 \| 15.00 \| no data \| |  |  |  |
| --- | --- | --- | --- | --- | --- | --- | --- | --- | --- | --- | --- | --- | --- | --- | --- | --- | --- | --- | --- | --- | --- | --- | --- | --- | --- | --- | --- | --- | --- | --- | --- | --- | --- | --- | --- | --- | --- | --- | --- | --- | --- | --- | --- | --- | --- | --- | --- | --- | --- | --- | --- | --- | --- | --- | --- | --- | --- | --- |
|  | | |  |

**Supplement 2c: Test data for 16 patients who were ultimately not included in the study (NoNIP) compared with 10 patients who were included (NIP).**

| **StudyNo** | **TICS Total Score** | **GDS Total Score** | **Preop Word List Total 3 Trials** | **Preop Verbal Fluency Total Correct, Letter 1** | **Preop Verbal Fluency Total Correct, Letter 2** | **Preop Digit Symbol Score** |  |
| --- | --- | --- | --- | --- | --- | --- | --- |
| NIP-1 |  |  | 21 |  |  | 26 |  |
| NIP-3 | 33.7 | 2 | 21 | 11 | 6 | 44 |  |
| NIP-4 | 35.6 | 1 | 19 | 6 | 17 | 46 |  |
| NIP-5 | 33.4 | 1 | 16 | 16 | 13 | 41 |  |
| NIP-6 | 24.2 | 0 | 10 | 4 | 4 | 20 |  |
| NIP-007 | 33.7 | 3 | 18 | 7 | 9 | 30 |  |
| NIP-008 | 35.6 | 4 | 18 | 15 | 16 | 40 |  |
| NIP-009 | 36.6 | 1 | 19 | 8 | 6 | 46 |  |
| NIP-010 | 32.2 | 2 | 14 | 15 | 16 | 55 |  |
| NIP-011 | 35.6 | 1 | 20 | 10 | 10 | 59 |  |
| *NoNIP-2* | *30.7* | *1* | *24* | *6* | *12* | *34* |  |
| *NoNIP-101* | *38.8* | *11* | *25* | *17* | *18* | *57* |  |
| *NoNIP-102* |  |  | *24* |  |  |  |  |
| *NoNIP-104* | *33.3* | *6* | *16* | *9* | *15* | *36* |  |
| *NoNIP-106* | *35.6* | *8* | *5* | *12* | *13* |  |  |
| *NoNIP-108* | *34.6* | *5* | *20* | *12* | *13* | *53* |  |
| *NoNIP-109* | *26.3* | *1* | *16* | *13* | *15* | *32* |  |
| *NoNIP-110* | *29.4* | *1* | *16* | *2* | *2* | *28* |  |
| *NoNIP-111* | *37.7* | *0* | *21* | *14* | *15* | *39* |  |
| *NoNIP-113* | *39.9* | *0* | *26* | *12* | *14* | *67* |  |
| *NoNIP-114* | *39.9* | *0* | *26* | *13* | *10* | *52* |  |
| *NoNIP-115* | *31.6* | *2* | *21* |  |  |  |  |
| *NoNIP-103* | *No cognitive tests done* | | | | | |  |
| *NoNIP-105* | *No cognitive tests done* | | | | | |  |
| *NoNIP-107* | *No cognitive tests done* | | | | | |  |
| *No NIP-112* | *No cognitive tests done* | | | | | |  |
|  |  |  |  |  |  |  |  |
| Average NIP | 33.40 | 1.67 | 17.60 | 10.22 | 10.78 | 40.70 |  |
| Average NoNIP | 34.35 | 3.18 | 20.00 | 11.00 | 12.70 | 44.22 |  |
| SD NIP | 3.73 | 1.22 | 3.44 | 4.35 | 4.92 | 12.32 |  |
| SD NoNIP | 4.53 | 3.76 | 6.06 | 4.29 | 4.32 | 13.38 |  |
| n NIP | 9 | 9 | 10 | 9 | 9 | 10 |  |
| n NoNIP | 11 | 11 | 12 | 10 | 10 | 9 |  |
| p ttest NIP vs. NoNIP | 0.6149 | 0.2319 | 0.2593 | 0.7005 | 0.3811 | 0.5601 |  |

Supplement 2d : Age and ASA scores for included and not included patients

| **StudyNo** | **Age** | **ASA** |
| --- | --- | --- |
| NIP-1 | 65 | 2 |
| NIP-3 | 84 | 2 |
| NIP-4 | 76 | 2 |
| NIP-5 | 75 | 2 |
| NIP-6 | 74 | 2 |
| NIP-007 | 61 | 3 |
| NIP-008 | 64 | 2 |
| NIP-009 | 80 | 2 |
| NIP-010 | 63 | 2 |
| NIP-011 | 59 | 1 |
| NoNIP-2 | 76 |  |
| NoNIP-101 | 65 | 2 |
| NoNIP-102 | 64 | 2 |
| NoNIP-103 | 74 | 2 |
| NoNIP-104 | 79 | 2 |
| NoNIP-105 | 59 | 3 |
| NoNIP-106 | 69 | 3 |
| NoNIP-107 |  | no anesthesia evaluation |
| NoNIP-108 | 69 | 2 |
| NoNIP-109 | 57 | 3 |
| NoNIP-110 | 66 | no anesthesia evaluation |
| NoNIP-111 | 79 | 3 |
| No NIP-112 |  | no anesthesia evaluation |
| NoNIP-113 | 62 | 1 |
| NoNIP-114 | 56 | no anesthesia evaluation |
| NoNIP-115 | 53 | no anesthesia evaluation |
|  |  |  |
| Average NIP | 70.10 |  |
| Average NoNIP | 66.29 |  |
| SD NIP | 8.72 |  |
| SD NoNIP | 8.50 |  |
| n NIP | 10 |  |
| n NoNIP | 14 (of 16) |  |

|  |  |  |  |  |
| --- | --- | --- | --- | --- |
|  |  |  |  |  |
|  |  |  |  |  |
|  |  |  |  |  |

| Supplement 2e: Preoperative medication   \| NIP-001 \| wellbutrin sr (bupropion hcl) 150 mg po bid taken  methadone 10 mg po bid taken  percocet po prn  ambien (zolpidem) po daily at bedtime  vitamin e daily stop 5-7 days  vitamin a daily stop 5-7 days  zinc daily stop 5-7 days  copper daily stop 5-7 days  beta carotene daily stop 5-7 days  omega fish oil daily stop 5-7 days \| \| --- \| --- \| \| NIP-002 \| amlodipine 5mg po daily in the am taken  ditropan (oxybutynin) 5mg po prn  diovan hct (valsartan/hctz) 160/25mg po daily in the am not taken morning of  levothyroxine 125mcg po daily in the am taken  keppra (levetriacetam) 500mg, 3 tabs po bid taken  vitamin b complex po daily  lamictal (lamotrigine) 100mg po bid taken 2 at night  calcet (calcium + vit d) po daily in the pm  multivitamin po daily stop 5-7 days  folic acid 400mcg, 2 tabs po bid not taken morning of  flaxseed oil w/ omega 3 daily stop 5-7 days  prevacid (lansoprazole) 30mg po daily in the am taken  iron supplement po daily in the am not taken morning of  tylenol (acetaminophen) 500mg po prn \| \| NIP-003 \| azopt eye drops 1gtt ou (both eyes) bid  other medication 1gtt ou (both eyes) bid  combigan lumigan eye drops 1gtt ou (both eyes) daily at bedtime \| \| NIP-004 \| remicade (infliximab) intravenous other infusions q 2 months - for crohn's disease  vicodin 5/500 mg po prn  ambien (zolpidem) 5 mg po daily at bedtime  niacin 1,000 mg po daily in the am not taken morning of  iron 1 tab po daily in the am not taken morning of  vitamin d (calciferol) 1 cap po daily in the am stop 5-7 days  calcium 1,000 mg po daily in the am stop 5-7 days  vitamin b12 (cyanocoabalamin) 1 tab po daily in the am stop 5-7 days  glucosamine 1 tab po daily in the am stop 5-7 days  lutein supplement 1 tab po daily in the am stop 5-7 days  fish oil 1 tbsp po bid stop 5-7 days \| \| NIP-005 \| lipitor (atorvastatin) 10mg po daily at bedtime not taken morning of  methotrexate 20mg po once a week not taken morning of  multivitamin folic acid, flax seed oil, b12 po daily not taken morning of  herbs fish oil po daily not taken morning of  aspirin 81mg po daily taken \| \| NIP-006 \| norvasc (amlodipine) 10 mg po daily in the pm  aspirin 81 mg po daily stop 5-7 days  voltaren (diclofenac) 75 mg, 2 tabs po daily stop 5-7 days  other medication lidex 0.05% cream topical bid  hctz 50 mg po daily in the am taken  metoprolol 100 mg po bid taken  potassium chloride 20 meq po daily  lipitor (atorvastatin) 40 mg po daily at bedtime  multivitamin po daily stop 5-7 days \| \| NIP-007 \| allopurinol (zyloprim) 300 mg tablet taken 1 tablet by mouth every day  amlodipine besylate (amlodipine oral) taken 10 mg by mouth daily.  aspirin 81 mg ec tablet: taken 81 mg by mouth daily.  atenolol oral  taken 50 mg by mouth daily.  dorzolamide-timolol (cosopt) 2-0.5 % ophthalmic solution placed 1 drop into both eyes 2 times daily.  hydrochlorothiazide (hydrodiuril) 12.5 mg tablet taken 1 tablet (12.5 mg total) by mouth daily.  latanoprost (xalatan) 0.005 % ophthalmic solution placed 1 drop in each eye every night at bedtime  lidocaine (lidoderm) 5 %(700 mg/patch) patch placed 1 patch onto the skin once a day once a day. applied to affected area for up to 12 hours (then taken off for at least 12 hours).  lovastatin (mevacor) 40 mg tablet taken 1 tablet by mouth atbedtime  olmesartan medoxomil (benicar oral) taken 40 mg by mouth nightly at bedtime.  omega-3 fatty acids 1,000 mg capsule taken 2 g by mouth daily.  spironolactone (aldactone) 25 mg tablet taken 1 tablet (25 mg total) by mouth daily.  tekturna 300 mg tablet  taken 1 tablet (300 mg total) by mouth daily.  triamcinolone (kenalog) 0.1 % cream  applied twice daily as needed  hydrocodone-acetaminophen (norco) 5-325 mg per tablet taken 1-2 tablets by mouth every 6 (six) hours as needed for pain.  ibuprofen (advil,motrin) 400 mg tablet: taken 400 mg by mouth every 6 (six) hours as needed. \| \| NIP-008 \| albuterol inh  inhale 2 puffs into the lungs every 4 (four) hours as needed.  ascorbic acid (vitamin c) 1,000 mg tablet  taken 1,000 mg by mouth daily.  aspirin 81 mg ec tablet  taken 81 mg by mouth daily.  calcium carbonate 500 mg calcium (1,250 mg) capsule:taken 1,000 mg by mouth daily.  clonazepam (klonopin oral):taken 0.5 mg by mouth 3 (three) times daily as needed.  dietary supplement oral:taken by mouth. tumeric 1 tablet bid, krill oil 1 capsule qd, red algae 1 capsule daily, astral essence 2 tabs in am and 1 tabl in pm.  ergocalciferol, vitamin d2, (vitamin d oral):taken 5,000 mg by mouth daily.  fluoxetine hcl (prozac oral):taken 60 mg by mouth nightly at bedtime.  fluticasone (flovent hfa) 220 mcg/actuation inhaler:inhale 1 puff into the lungs nightly at bedtime.  ibuprofen (advil oral):taken 600 mg by mouth 2 (two) times daily.  lamotrigine (lamictal oral):taken 150 mg by mouth 2 (two) times daily.  liothyronine sodium (cytomel oral):taken 0.5 mcg by mouth daily.  lisinopril oral:taken 40 mg by mouth nightly at bedtime.  multivitamin tablet:taken 1 tablet by mouth daily.  zolpidem (ambien) 10 mg tablet taken 10 mg by mouth nightly as needed. \| \| NIP-009 \| aspirin 81 mg ec tablet taken 1 tablet (81 mg total) by mouth daily.  benazepril (lotensin) 20 mg tablet taken 1 tablet (20 mg total) by mouth daily.  calcium carbonate-vitd3 200 (500)-400 mg-unit taken 1 tablet by mouth daily.  cholecalciferol, vitamin d3 1000 units tab taken 1 tablet (1,000 units total) by mouth daily.  fluticasone (flonase) 50 mcg/actuation nasal spray  glimepiride (amaryl) 4 mg tablet taken 1 tablet (4 mg total) by mouth every morning before breakfast.  metformin (glucophage) 500 mg tablet taken 1,000 mg by mouth 2 (two) times daily with meals.  omeprazole (prilosec) 20 mg capsule taken 1 capsule (20 mg total) by mouth daily.  pioglitazone (actos) 30 mg tablet taken 1 tablet (30 mg total) by mouth daily.  rosuvastatin (crestor) 20 mg tablet taken 1 tablet (20 mg total) by mouth daily.  tamsulosin (flomax) 0.4 mg 24 hr capsule taken 0.4 mg by mouth daily.  benazepril (lotensin) 40 mg tablet taken 1 tablet (40 mg total) by mouth daily.  econazole nitrate 1 % cream applied twice daily as directed \| \| NIP-010 \| multivitamin tablet 1 tablet daily  senna (senokot) tablet 17.2 mg bedtime  ferrous sulfate tablet 325 mg 3 times daily with meals  amlodipine (norvasc) tablet 10 mg daily  atorvastatin (lipitor) tablet 10 mg every evening \| \| NIP-011 \| ergocalciferol, vitamin d2, (vitamin d oral) taken by mouth.  magnesium gluconate oral taken by mouth.  simvastatin (zocor) 40 mg tablet taken 40 mg by mouth daily. \| |  | |  | |  | |  |  |
| --- | --- | --- | --- | --- | --- | --- | --- | --- | --- | --- | --- | --- | --- | --- | --- | --- | --- | --- | --- | --- | --- | --- | --- | --- | --- | --- | --- | --- | --- | --- |
|  |  | |  | |  | |  |  |
|  |  | |  | |  | |  |  |
|  |  | |  | |  | |  |  |
|  |  | |  | |  | |  |  |
|  |  | |  | |  | |  |  |
|  |  | |  | |  | |  |  |
|  |  | |  | |  | |  |  |
|  |  | |  | |  | |  |  |
|  |  | |  | |  | |  |  |
|  |  | |  | |  | |  |  |
|  |  | |  | |  | |  |  |
|  |  | |  | |  | |  |  |
|  |  | |  | |  | |  |  |
|  |  | |  | |  | |  |  |
|  |  | |  | |  | |  |  |
|  |  | |  | |  | |  |  |
|  |  | |  | |  | |  |  |
|  |  | |  | |  | |  |  |
|  |  | |  | |  | |  |  |
|  |  | |  | |  | |  |  |
|  | |  | |  | |  | | |
|  |  | |  | |  | |  |  |
|  |  | |  | |  | |  |  |
|  |  | |  | |  | |  |  |
|  |  | |  | |  | |  |  |
|  |  | |  | |  | |  |  |
|  |  | |  | |  | |  |  |
|  |  | |  | |  | |  |  |
|  |  | |  | |  | |  |  |
|  |  | |  | |  | |  |  |
