# Supplementary material for: Perioperative cerebrospinal fluid and plasma inflammatory markers after orthopedic surgery
Source: J Neuroinflammation. 2016 Aug 30;13(1):211. doi: 10.1186/s12974-016-0681-9 (PMC5006595; doi:10.1186/s12974-016-0681-9)
Supplement: Additional file 4: — Cytokine detailed statistical results. (DOCX 104 kb) [file 12974_2016_681_MOESM4_ESM.docx]

Detailed statistical results

- Results in tables with borders indicate a statistically significant time trend of the postoperative change in cytokine content compared to the pre-operative measurement; results in tables without borders indicate that there is no statistically significant trend. All measurement values are in pg ml^-1^.

Plasma Amyloid β40

| **Solution for Fixed Effects** | | | | | |
| --- | --- | --- | --- | --- | --- |
| **Effect** | **Estimate** | **Standard Error** | **DF** | **t Value** | **Pr > \|t\|** |
| **Intercept** | -6.7427 | 6.7713 | 10.5 | -1.00 | 0.3418 |
| **TIME** | 0.4550 | 0.3064 | 19 | 1.48 | 0.1540 |

| **Estimates** | | | | | |
| --- | --- | --- | --- | --- | --- |
| **Label** | **Estimate** | **Standard Error** | **DF** | **t Value** | **Pr > \|t\|** |
| **time 3 vs pre** | -6.7427 | 6.7713 | 10.5 | -1.00 | 0.3418 |
| **time 6 vs pre** | -5.3779 | 6.5815 | 9.36 | -0.82 | 0.4342 |
| **time 18 vs pre** | 0.08157 | 7.0764 | 12.3 | 0.01 | 0.9910 |

| **Covariance Parameter Estimates** | | | | | |
| --- | --- | --- | --- | --- | --- |
| **Cov Parm** | **Subject** | **Estimate** | **Standard Error** | **Z Value** | **Pr > Z** |
| **UN(1,1)** | **ID** | 385.28 | 200.62 | 1.92 | 0.0274 |
| **Residual** |  | 118.30 | 38.3824 | 3.08 | 0.0010 |

Plasma Amyloid β42

| **Null Model Likelihood Ratio Test** | | |
| --- | --- | --- |
| **DF** | **Chi-Square** | **Pr > ChiSq** |
| 1 | 7.04 | 0.0080 |

| **Solution for Fixed Effects** | | | | | |
| --- | --- | --- | --- | --- | --- |
| **Effect** | **Estimate** | **Standard Error** | **DF** | **t Value** | **Pr > \|t\|** |
| **Intercept** | -1.0900 | 2.1896 | 17.3 | -0.50 | 0.6249 |
| **TIME** | -1.3735 | 0.8689 | 18 | -1.58 | 0.1314 |
| **TIME*TIME** | 0.09799 | 0.05414 | 18 | 1.81 | 0.0870 |

| **Estimates** | | | | | |
| --- | --- | --- | --- | --- | --- |
| **Label** | **Estimate** | **Standard Error** | **DF** | **t Value** | **Pr > \|t\|** |
| **time 3 vs pre** | -1.0900 | 2.1896 | 17.3 | -0.50 | 0.6249 |
| **time 6 vs pre** | -4.3285 | 2.1896 | 17.3 | -1.98 | 0.0642 |
| **time 18 vs pre** | 0.3550 | 2.1896 | 17.3 | 0.16 | 0.8731 |

| **Covariance Parameter Estimates** | | | | | |
| --- | --- | --- | --- | --- | --- |
| **Cov Parm** | **Subject** | **Estimate** | **Standard Error** | **Z Value** | **Pr > Z** |
| **UN(1,1)** | **ID** | 25.3340 | 15.6979 | 1.61 | 0.0533 |
| **Residual** |  | 22.6113 | 7.5371 | 3.00 | 0.0013 |

Plasma Calprotectin

| **Solution for Fixed Effects** | | | | | |
| --- | --- | --- | --- | --- | --- |
| **Effect** | **Estimate** | **Standard Error** | **DF** | **t Value** | **Pr > \|t\|** |
| **Intercept** | 1037.68 | 1151.60 | 9 | 0.90 | 0.3910 |
| **TIME** | 11.2549 | 113.39 | 9 | 0.10 | 0.9231 |

| **Estimates** | | | | | |
| --- | --- | --- | --- | --- | --- |
| **Label** | **Estimate** | **Standard Error** | **DF** | **t Value** | **Pr > \|t\|** |
| **time 3 vs pre** | 1037.68 | 1151.60 | 9 | 0.90 | 0.3910 |
| **time 6 vs pre** | 1071.44 | 840.78 | 9 | 1.27 | 0.2345 |
| **time 18 vs pre** | 1206.50 | 737.59 | 9 | 1.64 | 0.1363 |

| **Covariance Parameter Estimates** | | | | | |
| --- | --- | --- | --- | --- | --- |
| **Cov Parm** | **Subject** | **Estimate** | **Standard Error** | **Z Value** | **Pr Z** |
| **UN(1,1)** | **ID** | 8164938 | 6654193 | 1.23 | 0.1099 |
| **UN(2,1)** | **ID** | -832877 | 622014 | -1.34 | 0.1806 |
| **UN(2,2)** | **ID** | 63220 | 67283 | 0.94 | 0.1737 |
| **Residual** |  | 8233260 | 3682026 | 2.24 | 0.0127 |

Plasma IFNα2

| **Solution for Fixed Effects** | | | | | |
| --- | --- | --- | --- | --- | --- |
| **Effect** | **Estimate** | **Standard Error** | **DF** | **t Value** | **Pr > \|t\|** |
| **Intercept** | -0.4085 | 0.7211 | 17.1 | -0.57 | 0.5784 |
| **TIME** | -0.03852 | 0.05619 | 19 | -0.69 | 0.5013 |

| **Estimates** | | | | | |
| --- | --- | --- | --- | --- | --- |
| **Label** | **Estimate** | **Standard Error** | **DF** | **t Value** | **Pr > \|t\|** |
| **time 3 vs pre** | -0.4085 | 0.7211 | 17.1 | -0.57 | 0.5784 |
| **time 6 vs pre** | -0.5241 | 0.8888 | 17.7 | -0.59 | 0.5629 |
| **time 18 vs pre** | -0.9864 | 1.5615 | 18.6 | -0.63 | 0.5353 |

| **Covariance Parameter Estimates** | | | | | |
| --- | --- | --- | --- | --- | --- |
| **Cov Parm** | **Subject** | **Estimate** | **Standard Error** | **Z Value** | **Pr Z** |
| **UN(1,1)** | **ID** | 2.7374 | 1.0642 | 2.57 | 0.0051 |
| **UN(2,1)** | **ID** | 0.5921 | 0.1936 | 3.06 | 0.0022 |
| **UN(2,2)** | **ID** | 0 | . | . | . |
| **Residual** |  | 3.9784 | 1.2907 | 3.08 | 0.0010 |

Plasma IFNγ

| **Null Model Likelihood Ratio Test** | | |
| --- | --- | --- |
| **DF** | **Chi-Square** | **Pr > ChiSq** |
| 3 | 41.75 | <.0001 |

| **Solution for Fixed Effects** | | | | | |
| --- | --- | --- | --- | --- | --- |
| **Effect** | **Estimate** | **Standard Error** | **DF** | **t Value** | **Pr > \|t\|** |
| **Intercept** | -1.1024 | 0.7205 | 9 | -1.53 | 0.1604 |
| **TIME** | -0.06302 | 0.05952 | 9 | -1.06 | 0.3173 |

| **Estimates** | | | | | |
| --- | --- | --- | --- | --- | --- |
| **Label** | **Estimate** | **Standard Error** | **DF** | **t Value** | **Pr > \|t\|** |
| **time 3 vs pre** | -1.1024 | 0.7205 | 9 | -1.53 | 0.1604 |
| **time 6 vs pre** | -1.2914 | 0.8894 | 9 | -1.45 | 0.1805 |
| **time 18 vs pre** | -2.0477 | 1.5865 | 9 | -1.29 | 0.2290 |

| **Covariance Parameter Estimates** | | | | | |
| --- | --- | --- | --- | --- | --- |
| **Cov Parm** | **Subject** | **Estimate** | **Standard Error** | **Z Value** | **Pr Z** |
| **UN(1,1)** | **ID** | 3.2959 | 2.5894 | 1.27 | 0.1015 |
| **UN(2,1)** | **ID** | 0.5460 | 0.2061 | 2.65 | 0.0081 |
| **UN(2,2)** | **ID** | 0.01114 | 0.01992 | 0.56 | 0.2880 |
| **Residual** |  | 3.0605 | 1.3687 | 2.24 | 0.0127 |

Plasma IL-2

| **Solution for Fixed Effects** | | | | | |
| --- | --- | --- | --- | --- | --- |
| **Effect** | **Estimate** | **Standard Error** | **DF** | **t Value** | **Pr > \|t\|** |
| **Intercept** | -0.2500 | 0.2366 | 9 | -1.06 | 0.3183 |
| **TIME** | -0.01362 | 0.009302 | 9 | -1.46 | 0.1772 |

| **Estimates** | | | | | |
| --- | --- | --- | --- | --- | --- |
| **Label** | **Estimate** | **Standard Error** | **DF** | **t Value** | **Pr > \|t\|** |
| **time 3 vs pre** | -0.2500 | 0.2366 | 9 | -1.06 | 0.3183 |
| **time 6 vs pre** | -0.2908 | 0.2433 | 9 | -1.20 | 0.2625 |
| **time 18 vs pre** | -0.4542 | 0.2959 | 9 | -1.54 | 0.1591 |

| **Covariance Parameter Estimates** | | | | | |
| --- | --- | --- | --- | --- | --- |
| **Cov Parm** | **Subject** | **Estimate** | **Standard Error** | **Z Value** | **Pr Z** |
| **UN(1,1)** | **ID** | 0.5430 | 0.2640 | 2.06 | 0.0198 |
| **UN(2,1)** | **ID** | 0.005325 | 0.007480 | 0.71 | 0.4766 |
| **UN(2,2)** | **ID** | 0.000650 | 0.000419 | 1.55 | 0.0604 |
| **Residual** |  | 0.02710 | 0.01212 | 2.24 | 0.0127 |

Plasma IL-4

| **Solution for Fixed Effects** | | | | | |
| --- | --- | --- | --- | --- | --- |
| **Effect** | **Estimate** | **Standard Error** | **DF** | **t Value** | **Pr > \|t\|** |
| **Intercept** | -0.4179 | 1.2764 | 18.7 | -0.33 | 0.7470 |
| **TIME** | -0.1438 | 0.1257 | 19 | -1.14 | 0.2669 |

| **Estimates** | | | | | |
| --- | --- | --- | --- | --- | --- |
| **Label** | **Estimate** | **Standard Error** | **DF** | **t Value** | **Pr > \|t\|** |
| **time 3 vs pre** | -0.4179 | 1.2764 | 18.7 | -0.33 | 0.7470 |
| **time 6 vs pre** | -0.8493 | 1.0965 | 11.5 | -0.77 | 0.4543 |
| **time 18 vs pre** | -2.5748 | 1.5299 | 25.9 | -1.68 | 0.1044 |

| **Covariance Parameter Estimates** | | | | | |
| --- | --- | --- | --- | --- | --- |
| **Cov Parm** | **Subject** | **Estimate** | **Standard Error** | **Z Value** | **Pr > Z** |
| **UN(1,1)** | **ID** | 3.9620 | 5.4419 | 0.73 | 0.2333 |
| **Residual** |  | 19.9175 | 6.4621 | 3.08 | 0.0010 |

Plasma IL-5

| **Solution for Fixed Effects** | | | | | | |
| --- | --- | --- | --- | --- | --- | --- |
| **Effect** | **POCD_Delirium** | **Estimate** | **Standard Error** | **DF** | **t Value** | **Pr > \|t\|** |
| **Intercept** |  | 0.09376 | 0.2710 | 9.75 | 0.35 | 0.7367 |
| **TIME** |  | 0.1111 | 0.05368 | 9 | 2.07 | 0.0684 |
| **POCD_Delirium** | **0** | -0.7949 | 0.3941 | 8 | -2.02 | 0.0784 |
| **POCD_Delirium** | **1** | 0 | . | . | . | . |

| **Estimates** | | | | | |
| --- | --- | --- | --- | --- | --- |
| **Label** | **Estimate** | **Standard Error** | **DF** | **t Value** | **Pr > \|t\|** |
| **time 3 vs pre grp1** | 0.09376 | 0.2710 | 9.75 | 0.35 | 0.7367 |
| **time 3 vs pre grp0** | -0.7012 | 0.3232 | 10 | -2.17 | 0.0552 |
| **time 6 vs pre grp1** | 0.4271 | 0.2360 | 8.09 | 1.81 | 0.1076 |
| **time 6 vs pre grp0** | -0.3678 | 0.2946 | 8.09 | -1.25 | 0.2467 |
| **time 18 vs pre grp1** | 1.7605 | 0.7096 | 9.63 | 2.48 | 0.0333 |
| **time 18 vs pre grp0** | 0.9656 | 0.7312 | 10.5 | 1.32 | 0.2148 |

| **Covariance Parameter Estimates** | | | | | |
| --- | --- | --- | --- | --- | --- |
| **Cov Parm** | **Subject** | **Estimate** | **Standard Error** | **Z Value** | **Pr Z** |
| **UN(1,1)** | **ID** | 0.3061 | 0.2504 | 1.22 | 0.1108 |
| **UN(2,1)** | **ID** | -0.05893 | 0.04725 | -1.25 | 0.2123 |
| **UN(2,2)** | **ID** | 0.02651 | 0.01362 | 1.95 | 0.0258 |
| **Residual** |  | 0.2901 | 0.1297 | 2.24 | 0.0127 |

If the group effect is ignored, the results for all patients are:

| **Estimates** | | | | | |
| --- | --- | --- | --- | --- | --- |
| **Label** | **Estimate** | **Standard Error** | **DF** | **t Value** | **Pr > \|t\|** |
| **time 3 vs pre** | -0.2242 | 0.2327 | 9 | -0.96 | 0.3605 |
| **time 6 vs pre** | 0.1091 | 0.2141 | 9 | 0.51 | 0.6225 |
| **time 18 vs pre** | 1.4426 | 0.7288 | 9 | 1.98 | 0.0791 |

Plasma IL-6

| **Solution for Fixed Effects** | | | | | |
| --- | --- | --- | --- | --- | --- |
| **Effect** | **Estimate** | **Standard Error** | **DF** | **t Value** | **Pr > \|t\|** |
| **Intercept** | 24.3380 | 36.8353 | 23.9 | 0.66 | 0.5151 |
| **TIME** | 62.8366 | 18.3620 | 18 | 3.42 | 0.0030 |
| **TIME*TIME** | -3.0634 | 1.1441 | 18 | -2.68 | 0.0154 |

| **Estimates** | | | | | |
| --- | --- | --- | --- | --- | --- |
| **Label** | **Estimate** | **Standard Error** | **DF** | **t Value** | **Pr > \|t\|** |
| **time 3 vs pre** | 24.3380 | 36.8353 | 23.9 | 0.66 | 0.5151 |
| **time 6 vs pre** | 185.28 | 36.8353 | 23.9 | 5.03 | <.0001 |
| **time 18 vs pre** | 277.62 | 36.8353 | 23.9 | 7.54 | <.0001 |

| **Covariance Parameter Estimates** | | | | | |
| --- | --- | --- | --- | --- | --- |
| **Cov Parm** | **Subject** | **Estimate** | **Standard Error** | **Z Value** | **Pr > Z** |
| **UN(1,1)** | **ID** | 3470.34 | 3412.43 | 1.02 | 0.1546 |
| **Residual** |  | 10098 | 3366.03 | 3.00 | 0.0013 |

Plasma IL-8

| **Solution for Fixed Effects** | | | | | | |
| --- | --- | --- | --- | --- | --- | --- |
| **Effect** | **POCD_Delirium** | **Estimate** | **Standard Error** | **DF** | **t Value** | **Pr > \|t\|** |
| **Intercept** |  | 4.5740 | 1.6689 | 13.8 | 2.74 | 0.0161 |
| **TIME** |  | 2.8397 | 0.6005 | 18 | 4.73 | 0.0002 |
| **TIME*TIME** |  | -0.1527 | 0.03742 | 18 | -4.08 | 0.0007 |
| **POCD_Delirium** | **0** | -4.7425 | 2.2721 | 8 | -2.09 | 0.0703 |
| **POCD_Delirium** | **1** | 0 | . | . | . |  |

| **Estimates** | | | | | |
| --- | --- | --- | --- | --- | --- |
| **Label** | **Estimate** | **Standard Error** | **DF** | **t Value** | **Pr > \|t\|** |
| **time 3 vs pre grp1** | 4.5740 | 1.6689 | 13.8 | 2.74 | 0.0161 |
| **time 3 vs pre grp0** | -0.1685 | 1.9539 | 11.9 | -0.09 | 0.9327 |
| **time 6 vs pre grp1** | 11.7190 | 1.6689 | 13.8 | 7.02 | <.0001 |
| **time 6 vs pre grp0** | 6.9765 | 1.9539 | 11.9 | 3.57 | 0.0039 |
| **time 18 vs pre grp1** | 12.8170 | 1.6689 | 13.8 | 7.68 | <.0001 |
| **time 18 vs pre grp0** | 8.0745 | 1.9539 | 11.9 | 4.13 | 0.0014 |

| **Covariance Parameter Estimates** | | | | | |
| --- | --- | --- | --- | --- | --- |
| **Cov Parm** | **Subject** | **Estimate** | **Standard Error** | **Z Value** | **Pr > Z** |
| **UN(1,1)** | **ID** | 8.7894 | 6.3101 | 1.39 | 0.0818 |
| **Residual** |  | 10.8015 | 3.6005 | 3.00 | 0.0013 |

If the group effect is ignored, the results for all patients are:

| **Estimates** | | | | | |
| --- | --- | --- | --- | --- | --- |
| **Label** | **Estimate** | **Standard Error** | **DF** | **t Value** | **Pr > \|t\|** |
| **time 3 vs pre** | 2.6770 | 1.5560 | 16.7 | 1.72 | 0.1038 |
| **time 6 vs pre** | 9.8220 | 1.5560 | 16.7 | 6.31 | <.0001 |
| **time 18 vs pre** | 10.9200 | 1.5560 | 16.7 | 7.02 | <.0001 |

Plasma IL-10

| **Solution for Fixed Effects** | | | | | |
| --- | --- | --- | --- | --- | --- |
| **Effect** | **Estimate** | **Standard Error** | **DF** | **t Value** | **Pr > \|t\|** |
| **Intercept** | 8.7546 | 6.5749 | 28 | 1.33 | 0.1938 |
| **TIME** | 2.2279 | 0.7445 | 28 | 2.99 | 0.0057 |

| **Estimates** | | | | | |
| --- | --- | --- | --- | --- | --- |
| **Label** | **Estimate** | **Standard Error** | **DF** | **t Value** | **Pr > \|t\|** |
| **time 3 vs pre** | 8.7546 | 6.5749 | 28 | 1.33 | 0.1938 |
| **time 6 vs pre** | 15.4383 | 5.3165 | 28 | 2.90 | 0.0071 |
| **time 18 vs pre** | 42.1731 | 8.2565 | 28 | 5.11 | <.0001 |

| **Covariance Parameter Estimates** | | | | | |
| --- | --- | --- | --- | --- | --- |
| **Cov Parm** | **Subject** | **Estimate** | **Standard Error** | **Z Value** | **Pr > Z** |
| **Residual** |  | 698.33 | 186.64 | 3.74 | <.0001 |

Plasma IL-12 p70.

| **Null Model Likelihood Ratio Test** | | |
| --- | --- | --- |
| **DF** | **Chi-Square** | **Pr > ChiSq** |
| 1 | 9.34 | 0.0022 |

| **Solution for Fixed Effects** | | | | | |
| --- | --- | --- | --- | --- | --- |
| **Effect** | **Estimate** | **Standard Error** | **DF** | **t Value** | **Pr > \|t\|** |
| **Intercept** | -0.8868 | 0.6504 | 12 | -1.36 | 0.1978 |
| **TIME** | -0.00893 | 0.04038 | 19 | -0.22 | 0.8273 |

| **Estimates** | | | | | |
| --- | --- | --- | --- | --- | --- |
| **Label** | **Estimate** | **Standard Error** | **DF** | **t Value** | **Pr > \|t\|** |
| **time 3 vs pre** | -0.8868 | 0.6504 | 12 | -1.36 | 0.1978 |
| **time 6 vs pre** | -0.9135 | 0.6156 | 9.73 | -1.48 | 0.1695 |
| **time 18 vs pre** | -1.0207 | 0.7045 | 15.7 | -1.45 | 0.1670 |

| **Covariance Parameter Estimates** | | | | | |
| --- | --- | --- | --- | --- | --- |
| **Cov Parm** | **Subject** | **Estimate** | **Standard Error** | **Z Value** | **Pr > Z** |
| **UN(1,1)** | **ID** | 2.9582 | 1.7316 | 1.71 | 0.0438 |
| **Residual** |  | 2.0541 | 0.6664 | 3.08 | 0.0010 |

Plasma MCP-1

| **Solution for Fixed Effects** | | | | | |
| --- | --- | --- | --- | --- | --- |
| **Effect** | **Estimate** | **Standard Error** | **DF** | **t Value** | **Pr > \|t\|** |
| **Intercept** | 167.54 | 89.3328 | 13.8 | 1.88 | 0.0821 |
| **TIME** | 91.6519 | 28.5782 | 18 | 3.21 | 0.0049 |
| **TIME*TIME** | -6.1115 | 1.7807 | 18 | -3.43 | 0.0030 |

| **Estimates** | | | | | |
| --- | --- | --- | --- | --- | --- |
| **Label** | **Estimate** | **Standard Error** | **DF** | **t Value** | **Pr > \|t\|** |
| **time 3 vs pre** | 167.54 | 89.3328 | 13.8 | 1.88 | 0.0821 |
| **time 6 vs pre** | 387.49 | 89.3328 | 13.8 | 4.34 | 0.0007 |
| **time 18 vs pre** | 167.23 | 89.3328 | 13.8 | 1.87 | 0.0826 |

| **Covariance Parameter Estimates** | | | | | |
| --- | --- | --- | --- | --- | --- |
| **Cov Parm** | **Subject** | **Estimate** | **Standard Error** | **Z Value** | **Pr > Z** |
| **UN(1,1)** | **ID** | 55343 | 30056 | 1.84 | 0.0328 |
| **Residual** |  | 24461 | 8153.55 | 3.00 | 0.0013 |

Plasma MIP-1α

| **Solution for Fixed Effects** | | | | | |
| --- | --- | --- | --- | --- | --- |
| **Effect** | **Estimate** | **Standard Error** | **DF** | **t Value** | **Pr > \|t\|** |
| **Intercept** | 2.7725 | 1.0663 | 15 | 2.60 | 0.0201 |
| **TIME** | 0.001983 | 0.3735 | 18 | 0.01 | 0.9958 |
| **TIME*TIME** | -0.00666 | 0.02327 | 18 | -0.29 | 0.7780 |

| **Estimates** | | | | | |
| --- | --- | --- | --- | --- | --- |
| **Label** | **Estimate** | **Standard Error** | **DF** | **t Value** | **Pr > \|t\|** |
| **time 3 vs pre** | 2.7725 | 1.0663 | 15 | 2.60 | 0.0201 |
| **time 6 vs pre** | 2.7185 | 1.0663 | 15 | 2.55 | 0.0222 |
| **time 18 vs pre** | 1.3035 | 1.0663 | 15 | 1.22 | 0.2404 |

| **Covariance Parameter Estimates** | | | | | |
| --- | --- | --- | --- | --- | --- |
| **Cov Parm** | **Subject** | **Estimate** | **Standard Error** | **Z Value** | **Pr > Z** |
| **UN(1,1)** | **ID** | 7.1901 | 4.0727 | 1.77 | 0.0387 |
| **Residual** |  | 4.1789 | 1.3930 | 3.00 | 0.0013 |

Plasma MIP-1β

| **Null Model Likelihood Ratio Test** | | |
| --- | --- | --- |
| **DF** | **Chi-Square** | **Pr > ChiSq** |
| 1 | 15.50 | <.0001 |

| **Solution for Fixed Effects** | | | | | |
| --- | --- | --- | --- | --- | --- |
| **Effect** | **Estimate** | **Standard Error** | **DF** | **t Value** | **Pr > \|t\|** |
| **Intercept** | 9.2902 | 4.3242 | 10.8 | 2.15 | 0.0552 |
| **TIME** | -0.2789 | 0.2152 | 19 | -1.30 | 0.2104 |

| **Estimates** | | | | | |
| --- | --- | --- | --- | --- | --- |
| **Label** | **Estimate** | **Standard Error** | **DF** | **t Value** | **Pr > \|t\|** |
| **time 3 vs pre** | 9.2902 | 4.3242 | 10.8 | 2.15 | 0.0552 |
| **time 6 vs pre** | 8.4535 | 4.1772 | 9.44 | 2.02 | 0.0722 |
| **time 18 vs pre** | 5.1063 | 4.5588 | 13.1 | 1.12 | 0.2828 |

| **Covariance Parameter Estimates** | | | | | |
| --- | --- | --- | --- | --- | --- |
| **Cov Parm** | **Subject** | **Estimate** | **Standard Error** | **Z Value** | **Pr > Z** |
| **UN(1,1)** | **ID** | 150.88 | 80.5391 | 1.87 | 0.0305 |
| **Residual** |  | 58.3348 | 18.9263 | 3.08 | 0.0010 |

Plasma RAGE

| **Solution for Fixed Effects** | | | | | |
| --- | --- | --- | --- | --- | --- |
| **Effect** | **Estimate** | **Standard Error** | **DF** | **t Value** | **Pr > \|t\|** |
| **Intercept** | 58.4684 | 16.8987 | 9 | 3.46 | 0.0072 |
| **TIME** | 0.06026 | 2.3814 | 9 | 0.03 | 0.9804 |

| **Estimates** | | | | | |
| --- | --- | --- | --- | --- | --- |
| **Label** | **Estimate** | **Standard Error** | **DF** | **t Value** | **Pr > \|t\|** |
| **time 3 vs pre** | 58.4684 | 16.8987 | 9 | 3.46 | 0.0072 |
| **time 6 vs pre** | 58.6492 | 14.1150 | 9 | 4.16 | 0.0025 |
| **time 18 vs pre** | 59.3724 | 29.5749 | 9 | 2.01 | 0.0756 |

| **Covariance Parameter Estimates** | | | | | |
| --- | --- | --- | --- | --- | --- |
| **Cov Parm** | **Subject** | **Estimate** | **Standard Error** | **Z Value** | **Pr Z** |
| **UN(1,1)** | **ID** | 1835.35 | 1421.41 | 1.29 | 0.0983 |
| **UN(2,1)** | **ID** | -150.47 | 158.27 | -0.95 | 0.3418 |
| **UN(2,2)** | **ID** | 43.6290 | 27.3660 | 1.59 | 0.0554 |
| **Residual** |  | 1648.22 | 737.11 | 2.24 | 0.0127 |

Plasma TNF-α

| **Solution for Fixed Effects** | | | | | |
| --- | --- | --- | --- | --- | --- |
| **Effect** | **Estimate** | **Standard Error** | **DF** | **t Value** | **Pr > \|t\|** |
| **Intercept** | 0.4446 | 0.6277 | 12.3 | 0.71 | 0.4919 |
| **TIME** | 0.04562 | 0.04087 | 19 | 1.12 | 0.2782 |

| **Estimates** | | | | | |
| --- | --- | --- | --- | --- | --- |
| **Label** | **Estimate** | **Standard Error** | **DF** | **t Value** | **Pr > \|t\|** |
| **time 3 vs pre** | 0.4446 | 0.6277 | 12.3 | 0.71 | 0.4919 |
| **time 6 vs pre** | 0.5815 | 0.5907 | 9.82 | 0.98 | 0.3485 |
| **time 18 vs pre** | 1.1289 | 0.6849 | 16.5 | 1.65 | 0.1182 |

| **Covariance Parameter Estimates** | | | | | |
| --- | --- | --- | --- | --- | --- |
| **Cov Parm** | **Subject** | **Estimate** | **Standard Error** | **Z Value** | **Pr > Z** |
| **UN(1,1)** | **ID** | 2.6371 | 1.5902 | 1.66 | 0.0486 |
| **Residual** |  | 2.1044 | 0.6828 | 3.08 | 0.0010 |

Plasma.IL6/IL10 (calculated ratio)

| **Null Model Likelihood Ratio Test** | | |
| --- | --- | --- |
| **DF** | **Chi-Square** | **Pr > ChiSq** |
| 1 | 1.26 | 0.2624 |

| **Solution for Fixed Effects** | | | | | |
| --- | --- | --- | --- | --- | --- |
| **Effect** | **Estimate** | **Standard Error** | **DF** | **t Value** | **Pr > \|t\|** |
| **Intercept** | 0.8908 | 1.1881 | 17.4 | 0.75 | 0.4634 |
| **TIME** | 0.2726 | 0.1111 | 19 | 2.45 | 0.0240 |

| **Estimates** | | | | | |
| --- | --- | --- | --- | --- | --- |
| **Label** | **Estimate** | **Standard Error** | **DF** | **t Value** | **Pr > \|t\|** |
| **time 3 vs pre** | 0.8908 | 1.1881 | 17.4 | 0.75 | 0.4634 |
| **time 6 vs pre** | 1.7085 | 1.0383 | 11.1 | 1.65 | 0.1278 |
| **time 18 vs pre** | 4.9797 | 1.4027 | 24.7 | 3.55 | 0.0016 |

| **Covariance Parameter Estimates** | | | | | |
| --- | --- | --- | --- | --- | --- |
| **Cov Parm** | **Subject** | **Estimate** | **Standard Error** | **Z Value** | **Pr > Z** |
| **UN(1,1)** | **ID** | 4.4795 | 4.8585 | 0.92 | 0.1783 |
| **Residual** |  | 15.5656 | 5.0501 | 3.08 | 0.0010 |

Plasma.IL8/IL10 (calculated ratio)

| **Solution for Fixed Effects** | | | | | |
| --- | --- | --- | --- | --- | --- |
| **Effect** | **Estimate** | **Standard Error** | **DF** | **t Value** | **Pr > \|t\|** |
| **Intercept** | -2.9915 | 3.0547 | 9 | -0.98 | 0.3530 |
| **TIME** | -0.01052 | 0.009911 | 9 | -1.06 | 0.3160 |

| **Estimates** | | | | | |
| --- | --- | --- | --- | --- | --- |
| **Label** | **Estimate** | **Standard Error** | **DF** | **t Value** | **Pr > \|t\|** |
| **time 3 vs pre** | -2.9915 | 3.0547 | 9 | -0.98 | 0.3530 |
| **time 6 vs pre** | -3.0231 | 3.0516 | 9 | -0.99 | 0.3477 |
| **time 18 vs pre** | -3.1494 | 3.0419 | 9 | -1.04 | 0.3275 |

| **Covariance Parameter Estimates** | | | | | |
| --- | --- | --- | --- | --- | --- |
| **Cov Parm** | **Subject** | **Estimate** | **Standard Error** | **Z Value** | **Pr Z** |
| **UN(1,1)** | **ID** | 93.2931 | 43.9881 | 2.12 | 0.0170 |
| **UN(2,1)** | **ID** | -0.03185 | 0.1015 | -0.31 | 0.7538 |
| **UN(2,2)** | **ID** | 0.000727 | 0.000477 | 1.53 | 0.0636 |
| **Residual** |  | 0.03211 | 0.01436 | 2.24 | 0.0127 |

CSF Amyloid β42

| **Solution for Fixed Effects** | | | | | |
| --- | --- | --- | --- | --- | --- |
| **Effect** | **Estimate** | **Standard Error** | **DF** | **t Value** | **Pr > \|t\|** |
| **Intercept** | -80.9188 | 116.84 | 18.6 | -0.69 | 0.4972 |
| **TIME** | 19.6295 | 11.4610 | 19 | 1.71 | 0.1030 |

| **Estimates** | | | | | |
| --- | --- | --- | --- | --- | --- |
| **Label** | **Estimate** | **Standard Error** | **DF** | **t Value** | **Pr > \|t\|** |
| **time 3 vs pre** | -80.9188 | 116.84 | 18.6 | -0.69 | 0.4972 |
| **time 6 vs pre** | -22.0302 | 100.53 | 11.4 | -0.22 | 0.8304 |
| **time 18 vs pre** | 213.52 | 139.87 | 25.8 | 1.53 | 0.1390 |
| **Covariance Parameter Estimates** | | | | | |
| **Cov Parm** | **Subject** | **Estimate** | **Standard Error** | **Z Value** | **Pr > Z** |
| **UN(1,1)** | **ID** | 34069 | 45717 | 0.75 | 0.2281 |
| **Residual** |  | 165506 | 53697 | 3.08 | 0.0010 |

CSF Calprotectin

| **Solution for Fixed Effects** | | | | | |
| --- | --- | --- | --- | --- | --- |
| **Effect** | **Estimate** | **Standard Error** | **DF** | **t Value** | **Pr > \|t\|** |
| **Intercept** | 0.01370 | 3.1655 | 19 | 0.00 | 0.9966 |
| **TIME** | 2.0823 | 1.4241 | 8.98 | 1.46 | 0.1778 |

| **Estimates** | | | | | |
| --- | --- | --- | --- | --- | --- |
| **Label** | **Estimate** | **Standard Error** | **DF** | **t Value** | **Pr > \|t\|** |
| **time 3 vs pre** | 0.01370 | 3.1655 | 19 | 0.00 | 0.9966 |
| **time 6 vs pre** | 6.2605 | 3.8747 | 8.92 | 1.62 | 0.1409 |
| **time 18 vs pre** | 31.2478 | 20.0006 | 8.9 | 1.56 | 0.1530 |

| **Covariance Parameter Estimates** | | | | | |
| --- | --- | --- | --- | --- | --- |
| **Cov Parm** | **Subject** | **Estimate** | **Standard Error** | **Z Value** | **Pr Z** |
| **UN(1,1)** | **ID** | 0 | . | . | . |
| **UN(2,1)** | **ID** | -14.3906 | 14.4423 | -1.00 | 0.3190 |
| **UN(2,2)** | **ID** | 18.9953 | 9.5100 | 2.00 | 0.0229 |
| **Residual** |  | 161.86 | 52.5155 | 3.08 | 0.0010 |

CSF IFNα2

| **Solution for Fixed Effects** | | | | | |
| --- | --- | --- | --- | --- | --- |
| **Effect** | **Estimate** | **Standard Error** | **DF** | **t Value** | **Pr > \|t\|** |
| **Intercept** | -24.9032 | 38.3782 | 9.01 | -0.65 | 0.5326 |
| **TIME** | 0.5679 | 0.8916 | 9.01 | 0.64 | 0.5400 |

| **Estimates** | | | | | |
| --- | --- | --- | --- | --- | --- |
| **Label** | **Estimate** | **Standard Error** | **DF** | **t Value** | **Pr > \|t\|** |
| **time 3 vs pre** | -24.9032 | 38.3782 | 9.01 | -0.65 | 0.5326 |
| **time 6 vs pre** | -23.1995 | 35.8509 | 9.01 | -0.65 | 0.5337 |
| **time 18 vs pre** | -16.3843 | 26.0383 | 9.01 | -0.63 | 0.5448 |

| **Covariance Parameter Estimates** | | | | | |
| --- | --- | --- | --- | --- | --- |
| **Cov Parm** | **Subject** | **Estimate** | **Standard Error** | **Z Value** | **Pr Z** |
| **UN(1,1)** | **ID** | 14596 | 6940.91 | 2.10 | 0.0177 |
| **UN(2,1)** | **ID** | -314.39 | 157.22 | -2.00 | 0.0455 |
| **UN(2,2)** | **ID** | 6.2497 | 3.8221 | 1.64 | 0.0510 |
| **Residual** |  | 214.13 | 95.7610 | 2.24 | 0.0127 |

CSF IFNγ

| **Solution for Fixed Effects** | | | | | |
| --- | --- | --- | --- | --- | --- |
| **Effect** | **Estimate** | **Standard Error** | **DF** | **t Value** | **Pr > \|t\|** |
| **Intercept** | 0.8508 | 0.3273 | 14.8 | 2.60 | 0.0203 |
| **TIME** | -0.01488 | 0.02651 | 19 | -0.56 | 0.5811 |

| **Estimates** | | | | | |
| --- | --- | --- | --- | --- | --- |
| **Label** | **Estimate** | **Standard Error** | **DF** | **t Value** | **Pr > \|t\|** |
| **time 3 vs pre** | 0.8508 | 0.3273 | 14.8 | 2.60 | 0.0203 |
| **time 6 vs pre** | 0.8061 | 0.2969 | 10.4 | 2.71 | 0.0210 |
| **time 18 vs pre** | 0.6276 | 0.3725 | 21 | 1.68 | 0.1068 |

| **Covariance Parameter Estimates** | | | | | |
| --- | --- | --- | --- | --- | --- |
| **Cov Parm** | **Subject** | **Estimate** | **Standard Error** | **Z Value** | **Pr > Z** |
| **UN(1,1)** | **ID** | 0.5233 | 0.3975 | 1.32 | 0.0940 |
| **Residual** |  | 0.8853 | 0.2872 | 3.08 | 0.0010 |

CSF IL-2

| **Solution for Fixed Effects** | | | | | |
| --- | --- | --- | --- | --- | --- |
| **Effect** | **Estimate** | **Standard Error** | **DF** | **t Value** | **Pr > \|t\|** |
| **Intercept** | -0.01071 | 0.01036 | 28 | -1.03 | 0.3100 |
| **TIME** | 0.000536 | 0.001173 | 28 | 0.46 | 0.6515 |

| **Estimates** | | | | | |
| --- | --- | --- | --- | --- | --- |
| **Label** | **Estimate** | **Standard Error** | **DF** | **t Value** | **Pr > \|t\|** |
| **time 3 vs pre** | -0.01071 | 0.01036 | 28 | -1.03 | 0.3100 |
| **time 6 vs pre** | -0.00911 | 0.008380 | 28 | -1.09 | 0.2864 |
| **time 18 vs pre** | -0.00268 | 0.01301 | 28 | -0.21 | 0.8384 |

| **Covariance Parameter Estimates** | | | | | |
| --- | --- | --- | --- | --- | --- |
| **Cov Parm** | **Subject** | **Estimate** | **Standard Error** | **Z Value** | **Pr > Z** |
| **UN(1,1)** | **ID** | 0 | . | . | . |
| **Residual** |  | 0.001735 | 0.000464 | 3.74 | <.0001 |

CSF IL-4

The model cannot be estimated due to insufficient data

CSF IL-5

| **Solution for Fixed Effects** | | | | | |
| --- | --- | --- | --- | --- | --- |
| **Effect** | **Estimate** | **Standard Error** | **DF** | **t Value** | **Pr > \|t\|** |
| **Intercept** | -0.08800 | 0.04347 | 13.9 | -2.02 | 0.0626 |
| **TIME** | 0.04737 | 0.01407 | 18 | 3.37 | 0.0034 |
| **TIME*TIME** | -0.00234 | 0.000877 | 18 | -2.67 | 0.0155 |

| **Estimates** | | | | | |
| --- | --- | --- | --- | --- | --- |
| **Label** | **Estimate** | **Standard Error** | **DF** | **t Value** | **Pr > \|t\|** |
| **time 3 vs pre** | -0.08800 | 0.04347 | 13.9 | -2.02 | 0.0626 |
| **time 6 vs pre** | 0.03300 | 0.04347 | 13.9 | 0.76 | 0.4604 |
| **time 18 vs pre** | 0.09500 | 0.04347 | 13.9 | 2.19 | 0.0465 |

| **Covariance Parameter Estimates** | | | | | |
| --- | --- | --- | --- | --- | --- |
| **Cov Parm** | **Subject** | **Estimate** | **Standard Error** | **Z Value** | **Pr > Z** |
| **UN(1,1)** | **ID** | 0.01297 | 0.007075 | 1.83 | 0.0334 |
| **Residual** |  | 0.005931 | 0.001977 | 3.00 | 0.0013 |

CSF IL-6

| **Solution for Fixed Effects** | | | | | |
| --- | --- | --- | --- | --- | --- |
| **Effect** | **Estimate** | **Standard Error** | **DF** | **t Value** | **Pr > \|t\|** |
| **Intercept** | 36.6352 | 29.4802 | 18.2 | 1.24 | 0.2297 |
| **TIME** | 9.6395 | 2.8522 | 19 | 3.38 | 0.0031 |

| **Estimates** | | | | | |
| --- | --- | --- | --- | --- | --- |
| **Label** | **Estimate** | **Standard Error** | **DF** | **t Value** | **Pr > \|t\|** |
| **time 3 vs pre** | 36.6352 | 29.4802 | 18.2 | 1.24 | 0.2297 |
| **time 6 vs pre** | 65.5538 | 25.4840 | 11.3 | 2.57 | 0.0254 |
| **time 18 vs pre** | 181.23 | 35.1450 | 25.5 | 5.16 | <.0001 |

| **Covariance Parameter Estimates** | | | | | |
| --- | --- | --- | --- | --- | --- |
| **Cov Parm** | **Subject** | **Estimate** | **Standard Error** | **Z Value** | **Pr > Z** |
| **UN(1,1)** | **ID** | 2345.34 | 2933.81 | 0.80 | 0.2120 |
| **Residual** |  | 10250 | 3325.68 | 3.08 | 0.0010 |

CSF IL-8

| **Solution for Fixed Effects** | | | | | |
| --- | --- | --- | --- | --- | --- |
| **Effect** | **Estimate** | **Standard Error** | **DF** | **t Value** | **Pr > \|t\|** |
| **Intercept** | 47.9090 | 134.92 | 21.2 | 0.36 | 0.7260 |
| **TIME** | 170.20 | 61.8316 | 18 | 2.75 | 0.0131 |
| **TIME*TIME** | -9.6022 | 3.8527 | 18 | -2.49 | 0.0227 |

| **Estimates** | | | | | |
| --- | --- | --- | --- | --- | --- |
| **Label** | **Estimate** | **Standard Error** | **DF** | **t Value** | **Pr > \|t\|** |
| **time 3 vs pre** | 47.9090 | 134.92 | 21.2 | 0.36 | 0.7260 |
| **time 6 vs pre** | 472.10 | 134.92 | 21.2 | 3.50 | 0.0021 |
| **time 18 vs pre** | 440.46 | 134.92 | 21.2 | 3.26 | 0.0037 |

| **Covariance Parameter Estimates** | | | | | |
| --- | --- | --- | --- | --- | --- |
| **Cov Parm** | **Subject** | **Estimate** | **Standard Error** | **Z Value** | **Pr > Z** |
| **UN(1,1)** | **ID** | 67539 | 51429 | 1.31 | 0.0946 |
| **Residual** |  | 114503 | 38168 | 3.00 | 0.0013 |

CSF IL-10

| **Solution for Fixed Effects** | | | | | |
| --- | --- | --- | --- | --- | --- |
| **Effect** | **Estimate** | **Standard Error** | **DF** | **t Value** | **Pr > \|t\|** |
| **Intercept** | 12.6852 | 6.6524 | 14 | 1.91 | 0.0772 |
| **TIME** | 0.4084 | 0.5110 | 19 | 0.80 | 0.4341 |

| **Estimates** | | | | | |
| --- | --- | --- | --- | --- | --- |
| **Label** | **Estimate** | **Standard Error** | **DF** | **t Value** | **Pr > \|t\|** |
| **time 3 vs pre** | 12.6852 | 6.6524 | 14 | 1.91 | 0.0772 |
| **time 6 vs pre** | 13.9105 | 6.0994 | 10.2 | 2.28 | 0.0452 |
| **time 18 vs pre** | 18.8113 | 7.4837 | 19.8 | 2.51 | 0.0207 |

| **Covariance Parameter Estimates** | | | | | |
| --- | --- | --- | --- | --- | --- |
| **Cov Parm** | **Subject** | **Estimate** | **Standard Error** | **Z Value** | **Pr > Z** |
| **UN(1,1)** | **ID** | 238.85 | 168.11 | 1.42 | 0.0777 |
| **Residual** |  | 329.04 | 106.75 | 3.08 | 0.0010 |

CSF IL-12 p70

| **Solution for Fixed Effects** | | | | | |
| --- | --- | --- | --- | --- | --- |
| **Effect** | **Estimate** | **Standard Error** | **DF** | **t Value** | **Pr > \|t\|** |
| **Intercept** | -0.00700 | 0.1692 | 18.9 | -0.04 | 0.9674 |
| **TIME** | 0.1331 | 0.07155 | 18 | 1.86 | 0.0793 |
| **TIME*TIME** | -0.00813 | 0.004458 | 18 | -1.82 | 0.0847 |

| **Estimates** | | | | | |
| --- | --- | --- | --- | --- | --- |
| **Label** | **Estimate** | **Standard Error** | **DF** | **t Value** | **Pr > \|t\|** |
| **time 3 vs pre** | -0.00700 | 0.1692 | 18.9 | -0.04 | 0.9674 |
| **time 6 vs pre** | 0.3190 | 0.1692 | 18.9 | 1.89 | 0.0749 |
| **time 18 vs pre** | 0.1590 | 0.1692 | 18.9 | 0.94 | 0.3592 |

| **Covariance Parameter Estimates** | | | | | |
| --- | --- | --- | --- | --- | --- |
| **Cov Parm** | **Subject** | **Estimate** | **Standard Error** | **Z Value** | **Pr > Z** |
| **UN(1,1)** | **ID** | 0.1329 | 0.08841 | 1.50 | 0.0664 |
| **Residual** |  | 0.1533 | 0.05111 | 3.00 | 0.0013 |

CSF MCP-1

| **Solution for Fixed Effects** | | | | | |
| --- | --- | --- | --- | --- | --- |
| **Effect** | **Estimate** | **Standard Error** | **DF** | **t Value** | **Pr > \|t\|** |
| **Intercept** | 352.87 | 786.91 | 23.2 | 0.45 | 0.6580 |
| **TIME** | 1572.67 | 384.50 | 18 | 4.09 | 0.0007 |
| **TIME*TIME** | -93.7672 | 23.9580 | 18 | -3.91 | 0.0010 |

| **Estimates** | | | | | |
| --- | --- | --- | --- | --- | --- |
| **Label** | **Estimate** | **Standard Error** | **DF** | **t Value** | **Pr > \|t\|** |
| **time 3 vs pre** | 352.87 | 786.91 | 23.2 | 0.45 | 0.6580 |
| **time 6 vs pre** | 4226.98 | 786.91 | 23.2 | 5.37 | <.0001 |
| **time 18 vs pre** | 2845.31 | 786.91 | 23.2 | 3.62 | 0.0014 |

| **Covariance Parameter Estimates** | | | | | |
| --- | --- | --- | --- | --- | --- |
| **Cov Parm** | **Subject** | **Estimate** | **Standard Error** | **Z Value** | **Pr > Z** |
| **UN(1,1)** | **ID** | 1764343 | 1604769 | 1.10 | 0.1358 |
| **Residual** |  | 4427873 | 1475958 | 3.00 | 0.0013 |

CSF MIP-1α

| **Solution for Fixed Effects** | | | | | |
| --- | --- | --- | --- | --- | --- |
| **Effect** | **Estimate** | **Standard Error** | **DF** | **t Value** | **Pr > \|t\|** |
| **Intercept** | 14.5911 | 4.7278 | 9 | 3.09 | 0.0130 |
| **TIME** | -0.6721 | 0.3289 | 9 | -2.04 | 0.0714 |

| **Estimates** | | | | | |
| --- | --- | --- | --- | --- | --- |
| **Label** | **Estimate** | **Standard Error** | **DF** | **t Value** | **Pr > \|t\|** |
| **time 3 vs pre** | 14.5911 | 4.7278 | 9 | 3.09 | 0.0130 |
| **time 6 vs pre** | 12.5749 | 3.7649 | 9 | 3.34 | 0.0087 |
| **time 18 vs pre** | 4.5100 | 0.9706 | 9 | 4.65 | 0.0012 |

| **Covariance Parameter Estimates** | | | | | |
| --- | --- | --- | --- | --- | --- |
| **Cov Parm** | **Subject** | **Estimate** | **Standard Error** | **Z Value** | **Pr Z** |
| **UN(1,1)** | **ID** | 181.69 | 107.02 | 1.70 | 0.0448 |
| **UN(2,1)** | **ID** | -12.0347 | 7.4020 | -1.63 | 0.1040 |
| **UN(2,2)** | **ID** | 0.5458 | 0.5636 | 0.97 | 0.1664 |
| **Residual** |  | 67.5602 | 30.2139 | 2.24 | 0.0127 |

CSF MIP-1β

| **Solution for Fixed Effects** | | | | | |
| --- | --- | --- | --- | --- | --- |
| **Effect** | **Estimate** | **Standard Error** | **DF** | **t Value** | **Pr > \|t\|** |
| **Intercept** | 96.4536 | 24.6699 | 9 | 3.91 | 0.0036 |
| **TIME** | -5.4973 | 1.7467 | 9 | -3.15 | 0.0118 |

| **Estimates** | | | | | |
| --- | --- | --- | --- | --- | --- |
| **Label** | **Estimate** | **Standard Error** | **DF** | **t Value** | **Pr > \|t\|** |
| **time 3 vs pre** | 96.4536 | 24.6699 | 9 | 3.91 | 0.0036 |
| **time 6 vs pre** | 79.9618 | 19.5428 | 9 | 4.09 | 0.0027 |
| **time 18 vs pre** | 13.9946 | 4.9380 | 9 | 2.83 | 0.0196 |

| **Covariance Parameter Estimates** | | | | | |
| --- | --- | --- | --- | --- | --- |
| **Cov Parm** | **Subject** | **Estimate** | **Standard Error** | **Z Value** | **Pr Z** |
| **UN(1,1)** | **ID** | 5521.95 | 2880.06 | 1.92 | 0.0276 |
| **UN(2,1)** | **ID** | -380.17 | 202.34 | -1.88 | 0.0603 |
| **UN(2,2)** | **ID** | 23.2782 | 14.7417 | 1.58 | 0.0572 |
| **Residual** |  | 911.21 | 407.51 | 2.24 | 0.0127 |

CSF RAGE

| **Solution for Fixed Effects** | | | | | |
| --- | --- | --- | --- | --- | --- |
| **Effect** | **Estimate** | **Standard Error** | **DF** | **t Value** | **Pr > \|t\|** |
| **Intercept** | 0.1280 | 0.5769 | 12.1 | 0.22 | 0.8281 |
| **TIME** | -0.01150 | 0.03629 | 19 | -0.32 | 0.7548 |

| **Estimates** | | | | | |
| --- | --- | --- | --- | --- | --- |
| **Label** | **Estimate** | **Standard Error** | **DF** | **t Value** | **Pr > \|t\|** |
| **time 3 vs pre** | 0.1280 | 0.5769 | 12.1 | 0.22 | 0.8281 |
| **time 6 vs pre** | 0.09350 | 0.5453 | 9.76 | 0.17 | 0.8674 |
| **time 18 vs pre** | -0.04450 | 0.6262 | 15.9 | -0.07 | 0.9442 |

| **Covariance Parameter Estimates** | | | | | |
| --- | --- | --- | --- | --- | --- |
| **Cov Parm** | **Subject** | **Estimate** | **Standard Error** | **Z Value** | **Pr > Z** |
| **UN(1,1)** | **ID** | 2.3012 | 1.3575 | 1.70 | 0.0450 |
| **Residual** |  | 1.6597 | 0.5385 | 3.08 | 0.0010 |

CSF TNF-α

| **Solution for Fixed Effects** | | | | | |
| --- | --- | --- | --- | --- | --- |
| **Effect** | **Estimate** | **Standard Error** | **DF** | **t Value** | **Pr > \|t\|** |
| **Intercept** | 0.7233 | 0.3308 | 14.6 | 2.19 | 0.0455 |
| **TIME** | -0.01488 | 0.02651 | 19 | -0.56 | 0.5811 |

| **Estimates** | | | | | |
| --- | --- | --- | --- | --- | --- |
| **Label** | **Estimate** | **Standard Error** | **DF** | **t Value** | **Pr > \|t\|** |
| **time 3 vs pre** | 0.7233 | 0.3308 | 14.6 | 2.19 | 0.0455 |
| **time 6 vs pre** | 0.6786 | 0.3007 | 10.4 | 2.26 | 0.0467 |
| **time 18 vs pre** | 0.5001 | 0.3755 | 20.8 | 1.33 | 0.1974 |

| **Covariance Parameter Estimates** | | | | | |
| --- | --- | --- | --- | --- | --- |
| **Cov Parm** | **Subject** | **Estimate** | **Standard Error** | **Z Value** | **Pr > Z** |
| **UN(1,1)** | **ID** | 0.5460 | 0.4079 | 1.34 | 0.0903 |
| **Residual** |  | 0.8853 | 0.2872 | 3.08 | 0.0010 |

CSF.IL6/IL10 (calculated ratio)

| **Null Model Likelihood Ratio Test** | | |
| --- | --- | --- |
| **DF** | **Chi-Square** | **Pr > ChiSq** |
| 1 | 7.34 | 0.0068 |

| **Solution for Fixed Effects** | | | | | |
| --- | --- | --- | --- | --- | --- |
| **Effect** | **Estimate** | **Standard Error** | **DF** | **t Value** | **Pr > \|t\|** |
| **Intercept** | -6.4907 | 8.1123 | 12.6 | -0.80 | 0.4384 |
| **TIME** | 0.1882 | 0.5461 | 19 | 0.34 | 0.7342 |

| **Estimates** | | | | | |
| --- | --- | --- | --- | --- | --- |
| **Label** | **Estimate** | **Standard Error** | **DF** | **t Value** | **Pr > \|t\|** |
| **time 3 vs pre** | -6.4907 | 8.1123 | 12.6 | -0.80 | 0.4384 |
| **time 6 vs pre** | -5.9262 | 7.5998 | 9.89 | -0.78 | 0.4538 |
| **time 18 vs pre** | -3.6682 | 8.9011 | 17.1 | -0.41 | 0.6854 |

| **Covariance Parameter Estimates** | | | | | |
| --- | --- | --- | --- | --- | --- |
| **Cov Parm** | **Subject** | **Estimate** | **Standard Error** | **Z Value** | **Pr > Z** |
| **UN(1,1)** | **ID** | 425.46 | 262.77 | 1.62 | 0.0527 |
| **Residual** |  | 375.78 | 121.92 | 3.08 | 0.0010 |

CSF.IL8/IL10 (calculated ratio)

| **Null Model Likelihood Ratio Test** | | |
| --- | --- | --- |
| **DF** | **Chi-Square** | **Pr > ChiSq** |
| 1 | 5.86 | 0.0155 |

| **Solution for Fixed Effects** | | | | | |
| --- | --- | --- | --- | --- | --- |
| **Effect** | **Estimate** | **Standard Error** | **DF** | **t Value** | **Pr > \|t\|** |
| **Intercept** | 7.3300 | 34.4887 | 18.3 | 0.21 | 0.8340 |
| **TIME** | -28.1927 | 14.2922 | 18 | -1.97 | 0.0641 |
| **TIME*TIME** | 1.6252 | 0.8905 | 18 | 1.83 | 0.0846 |

| **Estimates** | | | | | |
| --- | --- | --- | --- | --- | --- |
| **Label** | **Estimate** | **Standard Error** | **DF** | **t Value** | **Pr > \|t\|** |
| **time 3 vs pre** | 7.3300 | 34.4887 | 18.3 | 0.21 | 0.8340 |
| **time 6 vs pre** | -62.6210 | 34.4887 | 18.3 | -1.82 | 0.0858 |
| **time 18 vs pre** | -49.8830 | 34.4887 | 18.3 | -1.45 | 0.1649 |

| **Covariance Parameter Estimates** | | | | | |
| --- | --- | --- | --- | --- | --- |
| **Cov Parm** | **Subject** | **Estimate** | **Standard Error** | **Z Value** | **Pr > Z** |
| **UN(1,1)** | **ID** | 5776.82 | 3746.73 | 1.54 | 0.0616 |
| **Residual** |  | 6117.85 | 2039.28 | 3.00 | 0.0013 |
